# Who is getting screened for diabetes according to body mass index and waist circumference categories in Peru? a pooled analysis of national surveys between 2015 and 2019

**DOI:** 10.1371/journal.pone.0256809

**Published:** 2021-08-27

**Authors:** Rodrigo M. Carrillo-Larco, Wilmer Cristobal Guzman-Vilca, Antonio Bernabe-Ortiz

**Affiliations:** 1 Department of Epidemiology and Biostatistics, School of Public Health, Imperial College London, London, United Kingdom; 2 CRONICAS Centre of Excellence in Chronic Diseases, Universidad Peruana Cayetano Heredia, Lima, Peru; 3 School of Medicine “Alberto Hurtado”, Universidad Peruana Cayetano Heredia, Lima, Peru; 4 Sociedad Científica de Estudiantes de Medicina Cayetano Heredia (SOCEMCH), Universidad Peruana Cayetano Heredia, Lima, Peru; 5 Universidad Científica del Sur, Lima, Peru; Medical University of Vienna, AUSTRIA

## Abstract

**Background:**

At the population level we would expect that people with obesity undergo diabetes screening tests more often than people with overweight and much more often than people with normal weight. We described the trends of diabetes screening according to body mass index (BMI) and waist circumference (WC) in Peru.

**Methods:**

Pooled analysis of health national surveys (2015–2019); men and women aged 35–70 years. We used relative frequencies to study: among those who have had a glucose test in the last year, how many there were in each BMI and WC category. We fitted a Poisson model to study whether people with high BMI or WC were more likely to have had a glucose test.

**Results:**

People with overweight (PR = 1.34; 95% CI: 1.29–1.38), obesity (PR = 1.57; 95% CI: 1.51–1.63) and central obesity (PR = 1.63; 95% CI: 1.35–1.96) were more likely to have had a glucose test. At the sub-national level, there was one (of twenty-five) region in which men with obesity were more often screened for diabetes than men with overweight and much more than men with normal weight. There were seven regions in which women with obesity were the most often screened for diabetes.

**Conclusions:**

Consistent with a risk-based prevention approach, people with obesity would be screened for diabetes more often than those with overweight and those with normal weight. This ideal profile was only observed in few regions. Diabetes screening strategies should be strengthened and homogenised, so that they reach those at high risk of diabetes.

## Introduction

Clinical guidelines recommend that diabetes screening should be informed by the risk factor profile of a person [1,2]. In other words, diabetes screening should follow a risk-based approach, whereby people at high risk (e.g., obese individuals) get screened aiming for an early diagnosis. Informing the decision of who requires a diabetes screening test, can be based on risk prediction equations [1–4], or on independent risk factors [1,2]. Major risk factors for diabetes are high body mass index (BMI) and waist circumference, often classified into overweight, obesity and central obesity. Therefore, and consistent with the risk-based approach considering a single risk factor, at the population level we would expect higher frequency of diabetes screening tests among people with obesity, followed by people with overweight, and finally those with normal weight. Assessing whether this pattern is observed at the national and sub-national levels, and whether there are time or geographic trends, could inform public health policies to strengthen diabetes screening programs. If there were a place where the largest proportion of diabetes screening tests were assigned to people with normal weight, their screening protocols could be revised to target people with higher risk. This could become a public health indicator to assess the allocation of glucose tests at the population level and sub-nationally.

We aimed to describe, at the population level, whether diabetes screening tests were most often applied to people with normal weight, overweight, obesity or central obesity; also, to quantify whether the probability of someone with high BMI or waist circumference to have had a glucose test in the last year improved between 2015 and 2019.

## Methods

### Study design

This is a pooled analysis of five (2015 to 2019) national surveys in Peru. The National Demographic and Health Survey (ENDES for its name in Spanish), follows a similar protocol as any other Demographic and Health Survey (DHS). The ENDES is conducted annually on a nationally representative sample of both men and women. In here, we pooled and analysed the last five ENDES surveys. These data are in the public domain [5].

The ENDES utilized a bietapic approach; thus, in rural areas, primary sampling units are clusters of 500–2,000 subjects, whereas in urban areas, these units are blocks or group of blocks with more than 2,000 subjects and an average of 140 households. In both cases, secondary sampling units comprise household within each of these clusters. Details of the sampling procedures are in technical documents of the ENDES [6].

### Study population

We studied a complete-case sample regarding BMI and having had a glucose test in the last year; that is, individuals with missing observations in these two variables were excluded. We did not apply any other selection criteria. We studied men and women aged between 35 and 70 years. As in any national health survey, the study population included a random sample of the general population.

### Variables

The main variable was whether the participant has had a glucose test in the last year; this was collected with a questionnaire: *In the last twelve months*, *has any physician or other health professional measured your glucose or blood sugar*? There were three options: Yes, No and Do not know; the last two options were combined into one and the variable coded as No versus Yes. The number of Do not know answers was very small (S1 Table).

BMI (kg/m^2^) was based on measured height and weight by trained fieldworkers. We classified this variable in three levels: normal weight (BMI <25 kg/m^2^), overweight (BMI ≥25 kg/m^2^ and BMI <30 kg/m^2^), and obesity (BMI ≥30 kg/m^2^).

Waist circumference (cm) was measured by trained fieldworkers as well, and we classified it into two groups: normal waist circumference or no central obesity (waist circumference <90 cm in men and <80 cm in women), and central obesity (waist circumference ≥90 cm in men and ≥80 cm in women) [7]. Waist circumference was introduced since 2018 (i.e., available only in 2018 and 2019).

Self-reported diabetes diagnosis was assessed with one question: *Have you ever been told by a physician that you have diabetes or high blood sugar*? There were three options: Yes, No and Do not know; the last two options were combined into one and the variable coded as No versus Yes. The number of Do not know answers was very small (S2 Table).

Information about the time elapsed since diabetes diagnosis and the survey year was available between 2015 and 2017. The question was: *How long ago were you told that you had diabetes or high blood sugar*?.

Finally, we also included age (in years) and sex of the participants; moreover, the analysis was presented at the sub-national level stratified by the twenty-five regions in Peru.

### Analysis

The statistical analyses were conduct with R (version 3.6.1) and STATA (version 16.1, College Station, Texas 77845, USA). The analysis code is available as supplementary material, along with datasets herein analysed.

#### Frequency of glucose tests by BMI categories

We aimed to describe the frequency of each BMI and waist circumference category among people who have had a glucose test in the previous year; in other words, among those who had a glucose test in the previous year (denominator), how many there were in the normal weight, overweight, obesity and central obesity categories. We computed these proportions by sex, year and region; these proportions were also computed at the national level by sex and year. This analysis accounted for the complex survey design of the ENDES. This analysis and the accompanying figures were developed in R.

#### Probability of having had a glucose test by BMI and waist circumference categories

Secondarily, we aimed to study whether people with overweight, obesity or central obesity had higher probability of having had a glucose test in the previous year, in comparison to people with normal weight. Moreover, we were interested in studying whether this probability changed throughout the observation period; that is, whether it would be more likely for someone with overweight, obesity or central obesity to have had a glucose test in 2019 than in 2015. To answer these questions, we developed a multi-level regression model of the Poisson family and link Log, including a random intercept (regions) and interaction terms between BMI or waist circumference categories and the study year (centred at 2017 because this was the midyear of the pooled surveys, i.e., study year minus 2017); the model also included age (in years) and sex as potential confounders. The outcome in the regression model was having had a glucose test in the previous year (no = 0 and yes = 1). We used the *meglm* command in STATA (S9 Table has the regression syntax).

#### Self-reported diabetes diagnosis among people who have had a glucose test by BMI category

This analysis was restricted to people who have been diagnosed with diabetes during the last twelve months or were free of diabetes, to be consistent with the question about having had a glucose test in the last year. The question we aimed to answer was: among those who had a glucose test in the last year and were also diagnosed (self-reported) with diabetes in the last year, how many people were there in each BMI category? Waist circumference was not included in this analysis because the surveys with waist circumference data did not have information about time since diabetes diagnosis. We used relative frequencies and this analysis accounted for the complex survey design of the ENDES. This analysis and the accompanying figures were developed in R.

### Ethics

This is a pooled analysis of de-identified survey data that are in the public domain [5]. We did not seek approval by an Institutional Review Board. The funders had no role in study design, data collection and analysis, decision to publish, or preparation of the manuscript. The authors are collectively responsible for the accuracy of the data. The arguments and opinions in this work are those of the authors alone, and do not represent the position of the institutions to which they belong.

## Results

### Study population

The pooled dataset included 75,333 people surveyed between 2015 and 2019; the contribution of each year to the overall sample size was virtually the same, ranging from 18.7% (2015) to 20.6% (2019). Overall, there were almost as many women (51.2%) as men (48.8%), and the mean age was 49.4 (95% confidence interval (95% CI): 49.2–49.5) years.

The overall frequency of BMI categories showed that almost half of the population had overweight (44.1%; 95% CI: 43.6%-44.7%), followed by obesity (28.9%; 95% CI: 28.4%-29.5%), and then normal weight (26.9%; 95% CI: 26.4%-27.5%). The proportion of people in the overweight and obesity categories increased since 2015 (Table 1). The proportion of central obesity in 2018 was 82.9% (95% CI: 82.0%-83.7%) and in 2019 it was 83.8% (95% CI: 83.0%-84.6%). Overall, 32.6% (95% CI: 32.0%-33.2%) of the population had a glucose test in the previous year; this proportion increased since 2015 (Table 2).

**Table 1:** Time trends of body mass index (BMI; kg/m^2^) categories at the national level.

Year	Normal weight (%)	Overweight (%)	Obesity (%)	Mean BMI
**2015 (n = 14,630)**	30.8 (29.7–32.0)	43.2 (42.1–44.3)	26.0 (24.9–27.1)	27.6 (27.4–27.7)
**2016 (n = 14,538)**	30.4 (29.2–31.5)	43.0 (41.8–44.2)	26.7 (25.6–27.8)	27.6 (27.5–27.8)
**2017 (n = 14,804)**	26.7 (25.6–27.9)	44.5 (43.3–45.8)	28.8 (27.5–30.1)	28.0 (27.9–28.1)
**2018 (n = 15,936)**	23.5 (22.5–24.6)	44.6 (43.4–45.9)	31.9 (30.6–33.1)	28.3 (28.2–28.4)
**2019 (n = 15,425)**	23.7 (22.7–24.8)	45.2 (44.0–46.5)	31.1 (29.9–32.3)	28.3 (28.2–28.4)

Categories are presented as prevalence estimates in percentages along with the 95% confidence interval. Mean BMI is also presented along with the 95% confidence interval.

**Table 2:** Time trends of self-reported glucose tests in the last year.

Year	Self-reported glucose test in the last year	
	No (%)	Yes (%)
**2015**	72.7 (70.4–72.9)	28.3 (27.1–29.5)
**2016**	70.2 (68.9–71.4)	29.8 (28.6–31.1)
**2017**	66.1 (64.7–67.5)	33.9 (32.6–35.3)
**2018**	66.0 (64.8–67.2)	34.0 (32.8–35.2)
**2019**	63.4 (62.2–64.7)	36.6 (35.3–37.8)

Categories are presented as prevalence estimates in percentages along with the 95% confidence interval.

Across years and comparing those who had a glucose test in the last year versus those who did not have a glucose test, the proportion of people with obesity was larger in the former group; this difference in the overweight stratum was less clear (S3 Table). Similarly, across years and comparing those who had a glucose test versus those who did not have a glucose test in the last year, the proportion of people with central obesity was larger in the former group (S4 Table)

### Frequency of glucose test by BMI categories–national results

**Fig 1:**
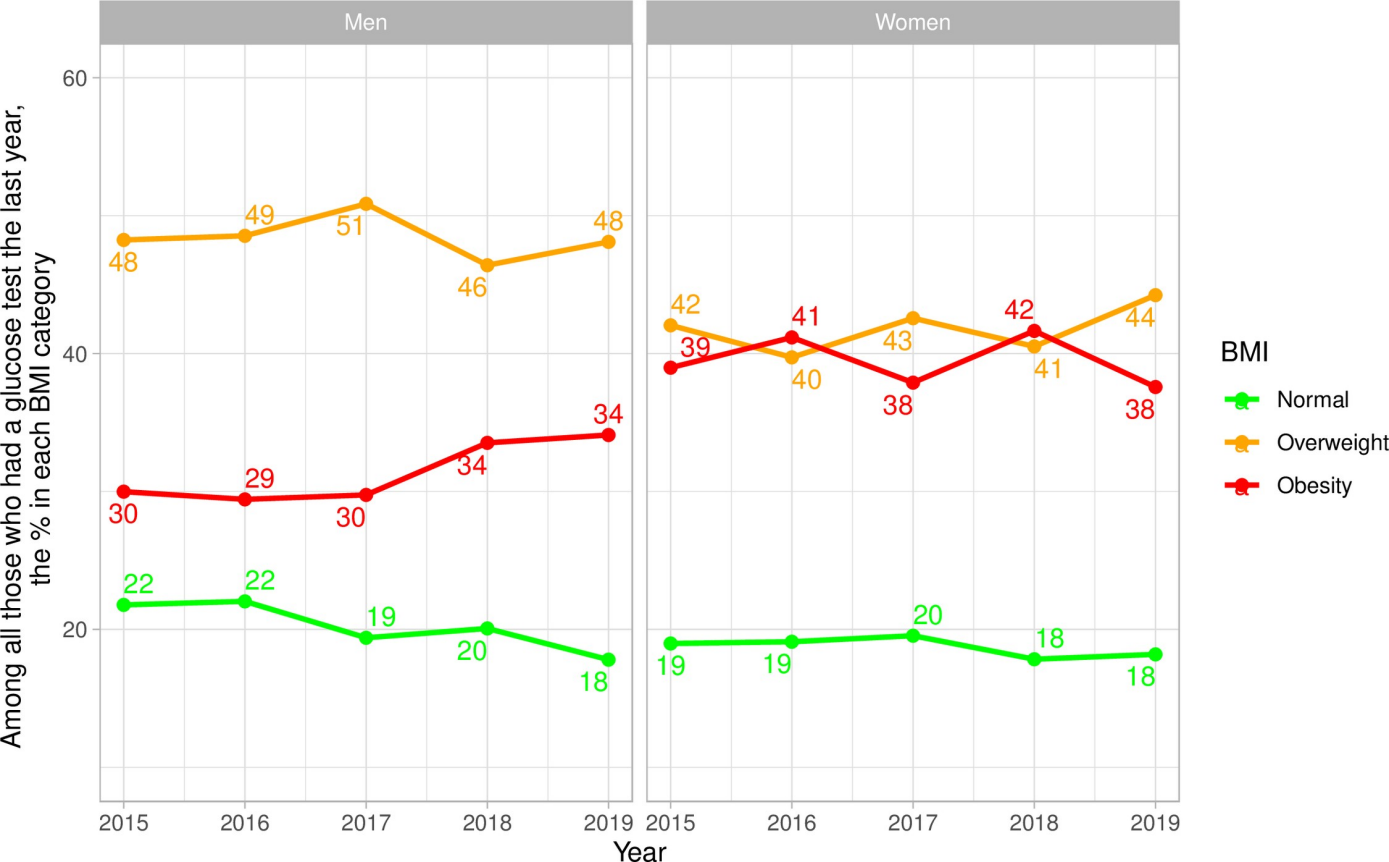
National time trends of glucose test by body mass index categories stratified by sex.

Nationally, among those who had a glucose test in the previous year, we observed that they were mostly in the overweight category across years for both men and women (Fig 1 and S5 Table). Specifically, among men who had a glucose test in the previous year, between 46% (2018) and 51% (2017) of these tests were in men with overweight, and between 29% (2016) and 34% (2019) were in men with obesity (Fig 1 and S5 Table). Among women who had a glucose test in the previous year, between 40% (2016) and 44% (2019) of these tests were in women with overweight; these numbers for women with obesity were 38% (2017 and 2019) and 42% (2018) (Fig 1 and S5 Table).

### Frequency of glucose test by waist circumference categories–national results

**Fig 2:**
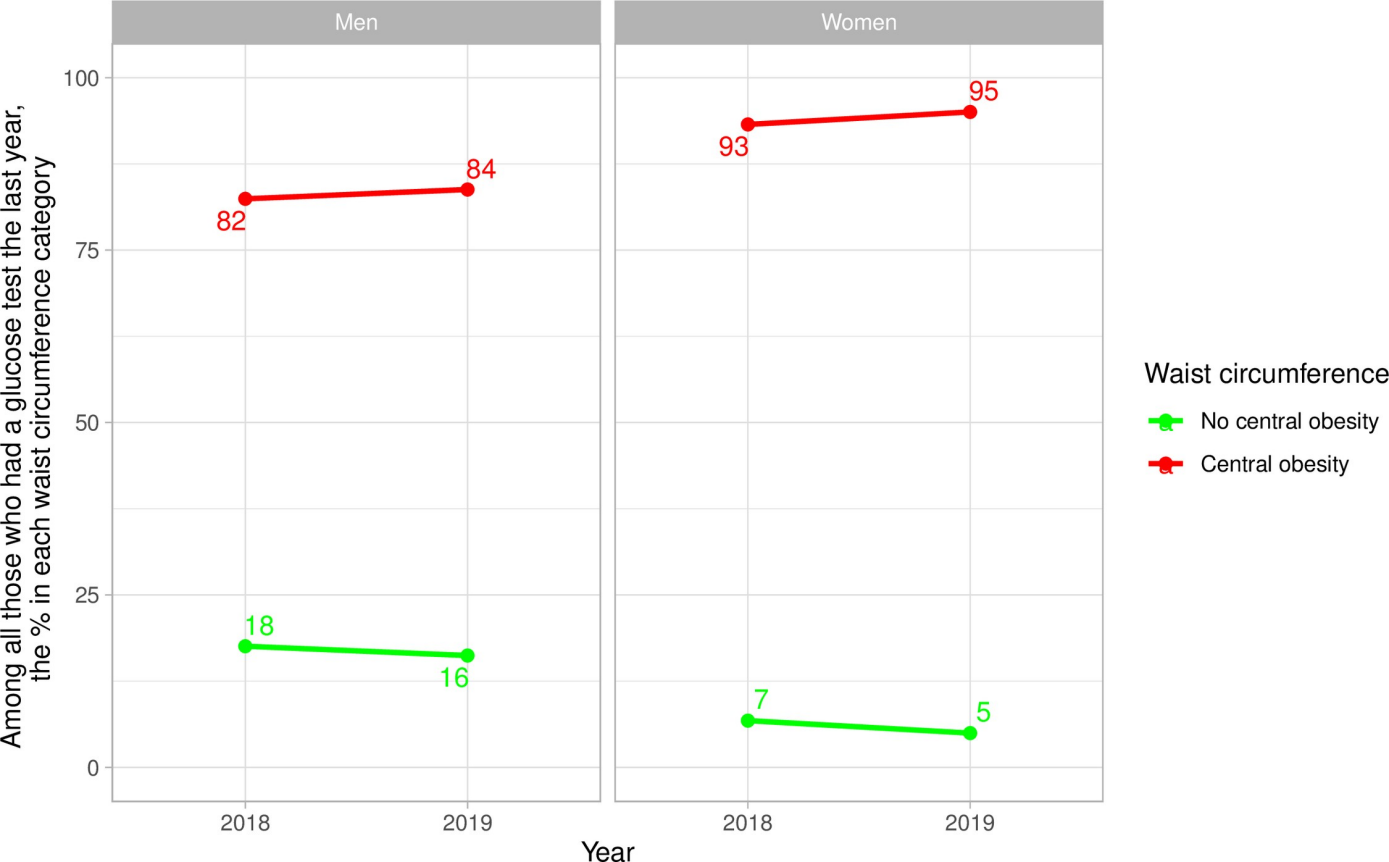
National time trends of glucose test by waist circumference categories stratified by sex.

People with central obesity were more often screened for diabetes (Fig 2 and S6 Table). In men, between 82% (2018) and 84% (2019) of those who had a glucose test the previous year were in the central obesity stratum; in women, these percentages were 93% (2018) and 95% (2019) (Fig 2 and S6 Table).

### Frequency of glucose test by BMI categories–sub-national results

Ideally, and following a risk-based approach, we would expect people with obesity to be most often screened with a glucose test, followed by people with overweight and then those in the normal weight range.

**Fig 3:**
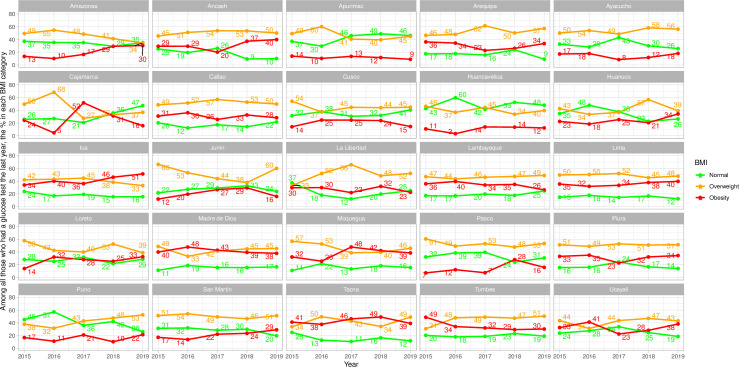
Sub-national time trends of glucose test by body mass index categories in men.

**Fig 4:**
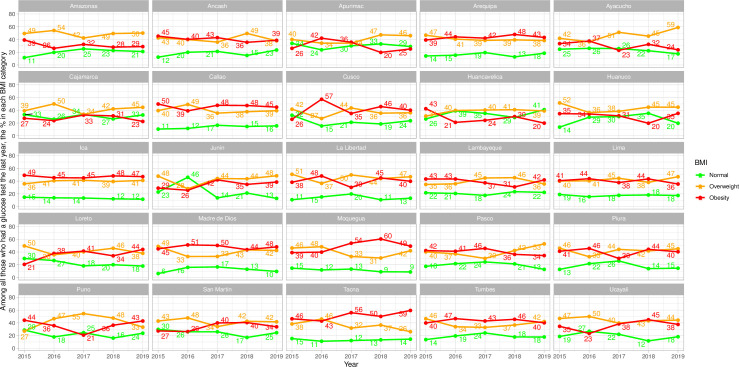
Sub-national time trends of glucose test by body mass index categories in women.

In men, this ideal pattern was observed in one (Ica) of the twenty-five regions (Fig 3 and S7 Table), and this pattern in this region arose in the last two years. In women, this ideal pattern was observed in one (Ica) of the twenty-five regions across the observation period; this ideal pattern emerged in six (Arequipa, Callao, Cusco, Madre de Dios, Moquegua and Tacna) additional regions (Fig 4 and S7 Table) at some point during the analysis period.

The exact opposite pattern was not found in any region throughout the full study period (Figs 3 and 4 and S7 Table). In men, the exact opposite pattern was found in one region (Apurimac) since 2016 and in another region (Huancavelica) since 2018 (Fig 3 and S7 Table). Women in the obesity group were the least screened in two regions (Apurimac and Huancavelica) in three years (Fig 4 and S7 Table).

### Frequency of glucose test by waist circumference categories–sub-national results

**Fig 5:**
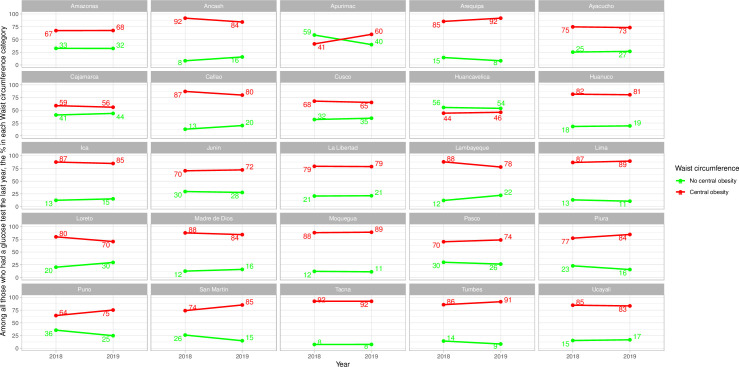
Sub-national time trends of glucose test by waist circumference categories in men.

**Fig 6:**
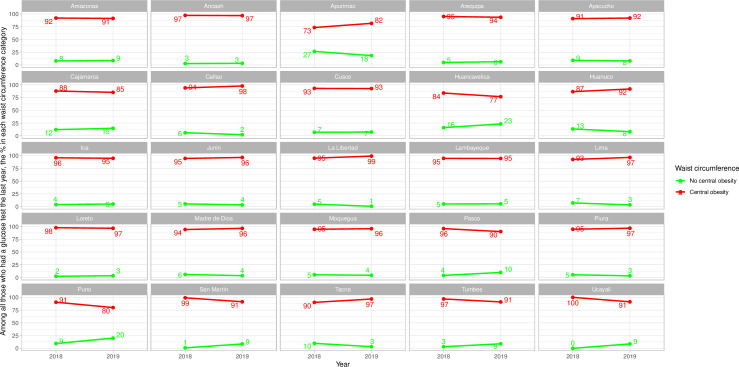
Sub-national time trends of glucose test by waist circumference categories in women.

Men and women in the central obesity category were always more often screened for diabetes (Figs 5 and 6 and S8 Table); this was not the case for men in Apurimac in 2018 and in Huancavelica in 2018 and 2019 (Fig 5 and S8 Table).

### Probability of having had a glucose test by BMI and waist circumference categories

The regression model revealed that, in comparison to people in the normal weight category, those with overweight (PR = 1.34; 95% CI: 1.29–1.38) and those with obesity (PR = 1.57; 95% CI: 1.51–1.63) were more likely to have had a glucose test the previous year (S9 Table). This likelihood appeared to have increased over the years (S9 Table). Similarly, people with central obesity were more likely to have had a glucose test the previous year, in comparison to their peers with waist circumference in the normal range (S9 Table). This likelihood did not appear to have improved over the years (S9 Table).

### Self-reported diabetes diagnosis among people who had a glucose test by BMI category

**Fig 7:**
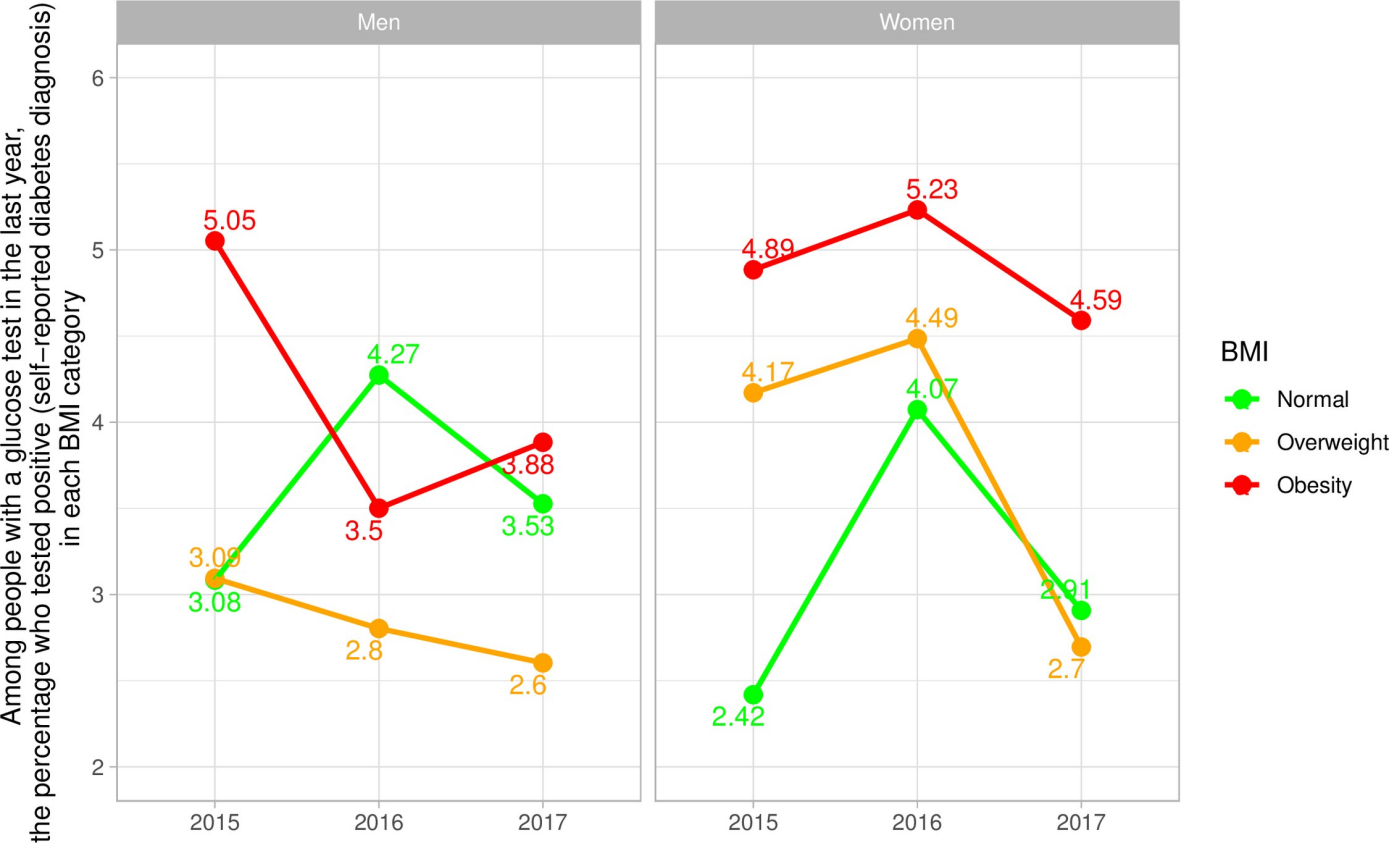
National trends of self-reported diabetes diagnosis among people who had a glucose test by body mass index category stratified by sex.

In women with obesity in 2019, among those who had a glucose test in the last year, almost 5% were then diagnosed with diabetes in the last twelve months; in other words, for every 100 women with obesity who had a glucose test in the previous year, 5 were then diagnosed with diabetes (Fig 7 and S10 Table). On the other hand, for every 100 women with normal weight who had a glucose test in the previous year, almost 3 were then diagnosed with diabetes (Fig 7 and S10 Table). We observed similar patterns in men, yet in smaller magnitude (Fig 7 and S10 Table).

## Discussion

### Main findings

In this pooled analysis of five national surveys in Peru between 2015 and 2019, we found that people with overweight, obesity and central obesity were more often screened for diabetes than their peers in the normal weight range. Consistent with this description, we observed that people with overweight, obesity and central obesity had higher probability of having had a glucose test in the previous year; notably, this probability has increased since 2015 for those with overweight or obesity. However, this ideal pattern of screening for diabetes among people with (central) obesity, followed by those with overweight and then those in the normal weight range, was not observed in all twenty-five regions. This ideal pattern was observed at some point in one region for men and in seven regions for women. Finally, we also observed that, among people who had a glucose test in the last year, there were more people diagnosed with diabetes (self-reported) in the obesity group. In general, there were no substantial differences between men and women. Overall, these findings signal regions where the diabetes screening protocols need to be revised, so that they mostly target people at high risk of diabetes because in this group we would most likely find undiagnosed diabetes cases.

### Public health implications

Ideally, we would screen for diabetes based on a stablished algorithm (e.g., risk prediction score [3,4]) or based on an assessment of risk factors (e.g., overweight plus first-degree relative with diabetes [2]). These approaches are suggested by international clinical guidelines [1,2]. In our analysis, we simplified this last approach by studying the glucose testing frequency stratified by BMI and waist circumference categories. The underlying rationale was that, the frequency of diabetes screening tests should be higher in people with (central) obesity, followed by people with overweight and then those in the normal weight range. This based on the fact that higher BMI is associated with higher diabetes risk [8]. This ideal scenario was found in a few regions, and this finding has pragmatic implications and could be introduced as a public health indicator to assess, at the population level, where diabetes screening tests are being applied to people at high risk.

The diabetes guidelines by the Peruvian Ministry of health reads: *we suggest screening with a fasting plasma glucose test in all adults aged 40–70 years who are obese or have overweight (…)* [9].Our results could suggest that this rule is being followed because we found that, generally, people with either obesity or overweight were more often screened than people with normal weight. Therefore, our work provides a simple monitoring framework to assess, at the population and sub-national level, where the suggested screening criteria are being followed and where this need consolidation.

Nonetheless, we did find some regions where people with normal weight would have a glucose test more often than people with overweight or obesity. In those regions, we would suggest strengthening the diabetes guidelines so that these are followed to the best of their ability and in accordance with the available resources. In addition, because people with obesity would have higher diabetes risk than people with overweight [8,10], we would suggest to reinforce the need for diabetes screening among obese individuals, similar to the ideal scenario we described above (more frequent diabetes screening in people with (central) obesity>overweight>normal weight).

Screening resources, unless unlimited, should be allocated where they are likely to find positive cases. Interestingly, all regions where we observed that people with overweigh or obesity were not often screened (e.g., even less often than people with normal weight) were in the Highlands. In the Highlands, overweight and obesity are less frequent than in other areas of Peru [11], therefore, although speculative, unhealthy BMI may not be seen as a risk factor for diabetes even though it has been acknowledged as such [12]. This hypothesis deserves further exploration to inform future media campaigns and other interventions. Moreover, these places in the Highlands are mostly rural and resource-limited, so screening tests may be restricted making it more relevant to target the population at high risk.

There were more regions showing the ideal scenario for women than for men. This agrees with the observation that women, in comparison to men, would be more likely to get tested for diabetes [13]. Furthermore, this could be explained by the fact that the prevalence of obesity is higher in women than in men in Peru [11,14]. Conversely, this finding could also suggest that men attend less often healthcare facilities, so they have fewer chances of getting screened for diabetes. It seems there is a need to improve diabetes screening among men. This observation is consistent with the differences there are between men and women regarding their diabetes profiles requiring sex-specific approaches [15,16]. We suggest improving diabetes screening opportunities for men. Perhaps, screening campaigns could be an option if it is with HbA1c (fasting not required). Although it seems that diabetes screening without any focalization does not lead to substantial long-term health gains [17], it may delay diabetes onset [18]. Alternatively, we could take advantage of all the opportunities. That is, whenever a man attends a healthcare centre for any reason, he could be assessed with a risk prediction equation [3,4] or based on his risk factor profile, and then invited to undergo a diabetes test as needed. Overall, results at the regional level suggested there were some sex differences, which should be acknowledged and incorporated in relevant policies so that men and women receive the best care according to their needs.

Our results also suggested that, among those who had a glucose test the previous year, there were more subsequent diabetes cases in the obesity group (4 out of 100 screenings in obese individuals). This would be equivalent to argue that screening people with obesity, assessed by BMI or waist circumference, has a pragmatic positive predictive value of 4% in diagnosing diabetes. A study in Peru showed the positive predictive value for four risk scores for undiagnosed diabetes ranged between 6% and 9% [19]. The difference in magnitude could be because we are using one risk factor only (high BMI), whereas risk scores ensemble multiple risk factors thus improving the accuracy. It seems that screening obese individuals would help to find new diabetes cases, and this could be further improved with the use of risk prediction scores.

In the current era of precision medicine, we argue that an absolute risk-based approach is the most convenient option. In other words, using risk prediction equations to compute the absolute risk based on several predictions would inform screening strategies more accurately. In this work, we opted for one risk factor only (i.e., high BMI or high waist circumference) because we aimed to provide a simple surveillance framework to monitor, at the population and sub-national level, where diabetes screening strategies warrant attention to ensure they are targeting those who need them the most.

The results herein summarised may not be directly applicable to other countries. The frequency of glucose tests in the general population according to BMI categories would depend on the screening strategies in each country (e.g., risk-based or test-all approach), on the rate of access to the healthcare system (e.g., universal health coverage), and on the BMI distribution in the population. Our results are not meant to inform other countries, but to spark interest in other countries to monitor the rates of glucose tests (i.e., diabetes screenings) according to variables that are often available in health surveys and that are closely related to the onset of diabetes (i.e., BMI and/or wait circumference). As we pointed out, this surveillance could help to identify places where the target population of glucose tests should be refined.

### Strengths and limitations

This analysis benefited from five recent national surveys in Peru. BMI and waist circumference were based on objective measurements, and all other variables were collected with a standard questionnaire and protocol. However, this work also has some limitations. First, although these were national surveys, some analysis could not be further stratified by region, because of limited number of observations. Limited data also excluded the possibility of analysis by age groups. Second, when studying diabetes diagnosis, this was based on self-reported information only, and not in a combination of self-reported diagnosis along with a biomarker (e.g., fasting glucose). The national surveys herein analysed do not collect any biomarkers. Future research, ideally other national surveys or large epidemiological studies should complement our results with more robust diagnosis criteria. Third, the rationale of our work was based on the argument that people with unhealthy BMI or waist circumference (i.e., overweight or obesity) would benefit from a glucose test. Nonetheless, a more comprehensive approach would be to use risk prediction equations which compute the absolute risk. We did not use this approach because of available data. Fourth, the information about having had a glucose test in the last year was self-reported, and we did not have further information on the reason why they had this test. There could be multiple reasons for a person in the general population to undergo a glucose test. Nonetheless, in the context of a national health survey, as part of a questionnaire looking at non-communicable diseases and risk factors, we argue that most of these glucose tests were meant for diabetes diagnosis. Readers are reminded to interpret the results in light of this limitation, which is shared by all national health surveys alike. Finally, we conducted a descriptive analysis which opened doors to further relevant research questions. For example, what would be the optimal frequency of glucose tests in the general population according to BMI and waist circumference to identify most or all diabetes cases? What be the effect of screening for diabetes all people with overweight or obesity in the general population? Also, are people with obesity not going to the doctor on a regular basis or are the doctors not screening people with obesity often? Descriptive analyses, such as ours, as the first step to study multiple questions.

## Conclusions

At the national level, glucose tests were most often allocated to people with overweight, obesity or central obesity. Unfortunately, this pattern was not consistent across all regions in Peru. This calls to strengthen and homogenise the diabetes screening criteria in all regions, so that the screening strategies target those who would benefit the most. Moreover, people with obesity and overweight who had a glucose test in the previous year, were more often diagnosed with diabetes. This supports the need to focus the screening resources to those at higher risk, thus allocating resources cost-effectively.

## Supporting information

S1 Table Number of observations in each possible answer to the question about self-reported glucose tests. (DOCX)

S2 Table Absolute number of observations in each possible answer to the question about self-reported diabetes diagnosis. (DOCX)

S3 Table Time trends of self-reported glucose tests in the last year by body mass index category. (DOCX)

S4 Table Time trends of self-reported glucose tests in the last year by waist circumference category. (DOCX)

S5 Table Frequency of glucose tests by body mass index categories at the national level. (DOCX)

S6 Table Frequency of glucose tests by waist circumference categories at the national level. (DOCX)

S7 Table Frequency of glucose tests by body mass index categories at the sub-national level. (DOCX)

S8 Table Frequency of glucose tests by waist circumference categories at the sub-national level. (DOCX)

S9 Table Regression models. (DOCX)

S10 Table Self-reported diabetes diagnosis among people who had a glucose test by BMI category. (DOCX)

S1 File (DO)

S2 File (R)

S3 File (CSV)

S4 File (CSV)

S5 File (CSV)

S6 File (CSV)

S7 File (CSV)
