# Supplementary material for: Who is getting screened for diabetes according to body mass index and waist circumference categories in Peru? a pooled analysis of national surveys between 2015 and 2019
Source: PLoS One. 2021 Aug 27;16(8):e0256809. doi: 10.1371/journal.pone.0256809 (PMC8396776; doi:10.1371/journal.pone.0256809)
Supplement: S1 Table — (DOCX) [file pone.0256809.s001.docx]

## **Supplementary table 1: number of observations in each possible answer to the question about self-reported glucose tests**

| **Year** | **Yes** | **No** | **Do not know** |
| --- | --- | --- | --- |
| **2015** | 3,929 | 10,687 | 14 |
| **2016** | 4,193 | 10,323 | 22 |
| **2017** | 4,434 | 10,342 | 28 |
| **2018** | 4,802 | 11,106 | 28 |
| **2019** | 4,863 | 10,522 | 40 |
