# Supplementary material for: Who is getting screened for diabetes according to body mass index and waist circumference categories in Peru? a pooled analysis of national surveys between 2015 and 2019
Source: PLoS One. 2021 Aug 27;16(8):e0256809. doi: 10.1371/journal.pone.0256809 (PMC8396776; doi:10.1371/journal.pone.0256809)
Supplement: S2 Table — (DOCX) [file pone.0256809.s002.docx]

## **Supplementary table 2: absolute number of observations in each possible answer to the question about self-reported diabetes diagnosis**

| **Year** | **Yes** | **No** | **Do not know** |
| --- | --- | --- | --- |
| **2015** | 804 | 32,086 | 16 |
| **2016** | 816 | 30,819 | 24 |
| **2017** | 865 | 31,619 | 30 |
| **2018** | 931 | 32,835 | 28 |
| **2019** | 966 | 31,922 | 33 |
