# Supplementary material for: Who is getting screened for diabetes according to body mass index and waist circumference categories in Peru? a pooled analysis of national surveys between 2015 and 2019
Source: PLoS One. 2021 Aug 27;16(8):e0256809. doi: 10.1371/journal.pone.0256809 (PMC8396776; doi:10.1371/journal.pone.0256809)
Supplement: S3 Table — (DOCX) [file pone.0256809.s003.docx]

## **Supplementary table 3: time trends of self-reported glucose tests in the last year by body mass index category**

|  | **Glucose test in the last year [N (%)]** | | | | | | | | | |
| --- | --- | --- | --- | --- | --- | --- | --- | --- | --- | --- |
| **Body mass index** | **2015** | | **2016** | | **2017** | | **2018** | | **2019** | |
|  | **No** | **Yes** | **No** | **Yes** | **No** | **Yes** | **No** | **Yes** | **No** | **Yes** |
| **Normal weight** | 3,804  (35.1) | 808  (20.2) | 3,650  (34.6) | 897  (20.4) | 3,481  (30.4) | 997  (19.5) | 3,432  (26.0) | 994  (18.8) | 3,253  (27.0) | 1,027  (18.0) |
| **Weight** | 4,516  (42.6) | 1,762  (44.7) | 4,373  (42.7) | 1,828  (43.6) | 4,464  (43.7) | 1,928  (46.3) | 4,847  (45.4) | 2,078  (43.1) | 4,612  (44.8) | 2,129  (45.9) |
| **Obesity** | 2,381  (22.4) | 1,359  (35.1) | 2,322  (22.7) | 1,468  (36.0) | 2,425  (25.9) | 1,509  (34.3) | 2,855  (28.6) | 1,730  (38.11) | 2,697  (28.2) | 1,707  (36.1) |
