# Supplementary material for: Who is getting screened for diabetes according to body mass index and waist circumference categories in Peru? a pooled analysis of national surveys between 2015 and 2019
Source: PLoS One. 2021 Aug 27;16(8):e0256809. doi: 10.1371/journal.pone.0256809 (PMC8396776; doi:10.1371/journal.pone.0256809)
Supplement: S4 Table — (DOCX) [file pone.0256809.s004.docx]

## **Supplementary table 4: time trends of self-reported glucose tests in the last year by waist circumference category**

|  | **Glucose test in the last year [N (%)]** | | | |
| --- | --- | --- | --- | --- |
| **Central obesity** | **2018** | | **2019** | |
|  | **No** | **Yes** | **No** | **Yes** |
| **No** | 2,714  (20.0) | 630  (11.5) | 2,516  (19.8) | 653  (9.9) |
| **Yes** | 8,293  (80.0) | 4,097  (88.5%) | 7,940  (80.2) | 4,132  (90.1) |
