# Supplementary material for: Who is getting screened for diabetes according to body mass index and waist circumference categories in Peru? a pooled analysis of national surveys between 2015 and 2019
Source: PLoS One. 2021 Aug 27;16(8):e0256809. doi: 10.1371/journal.pone.0256809 (PMC8396776; doi:10.1371/journal.pone.0256809)
Supplement: S5 Table — (DOCX) [file pone.0256809.s005.docx]

## **Supplementary table 5: frequency of glucose tests by body mass index categories at the national level**

| **Year** | **Sex** | **Normal weight** | **Normal weight lower limit** | **Normal weight upper limit** | **Overweight** | **Overweight lower limit** | **Overweight upper limit** | **Obesity** | **Obesity lower limit** | **Obesity upper limit** |
| --- | --- | --- | --- | --- | --- | --- | --- | --- | --- | --- |
| 2015 | Men | 0.2178 | 0.1910 | 0.2472 | 0.4824 | 0.4474 | 0.5175 | 0.2999 | 0.2690 | 0.3326 |
| 2016 | Men | 0.2203 | 0.1923 | 0.2512 | 0.4854 | 0.4505 | 0.5204 | 0.2943 | 0.2637 | 0.3269 |
| 2017 | Men | 0.1939 | 0.1691 | 0.2215 | 0.5086 | 0.4740 | 0.5431 | 0.2975 | 0.2637 | 0.3336 |
| 2018 | Men | 0.2007 | 0.1755 | 0.2285 | 0.4641 | 0.4289 | 0.4995 | 0.3353 | 0.3031 | 0.3690 |
| 2019 | Men | 0.1781 | 0.1554 | 0.2032 | 0.4810 | 0.4457 | 0.5164 | 0.3410 | 0.3088 | 0.3747 |
| 2015 | Women | 0.1898 | 0.1677 | 0.2140 | 0.4205 | 0.3920 | 0.4495 | 0.3898 | 0.3605 | 0.4198 |
| 2016 | Women | 0.1910 | 0.1699 | 0.2141 | 0.3972 | 0.3681 | 0.4271 | 0.4117 | 0.3840 | 0.4400 |
| 2017 | Women | 0.1954 | 0.1722 | 0.2210 | 0.4257 | 0.3963 | 0.4555 | 0.3789 | 0.3494 | 0.4094 |
| 2018 | Women | 0.1784 | 0.1578 | 0.2009 | 0.4052 | 0.3773 | 0.4336 | 0.4165 | 0.3892 | 0.4442 |
| 2019 | Women | 0.1819 | 0.1584 | 0.2080 | 0.4423 | 0.4115 | 0.4736 | 0.3758 | 0.3472 | 0.4052 |

Results are presents as proportions. Multiply these by 100 to get percentages.
