# Supplementary material for: Who is getting screened for diabetes according to body mass index and waist circumference categories in Peru? a pooled analysis of national surveys between 2015 and 2019
Source: PLoS One. 2021 Aug 27;16(8):e0256809. doi: 10.1371/journal.pone.0256809 (PMC8396776; doi:10.1371/journal.pone.0256809)
Supplement: S6 Table — (DOCX) [file pone.0256809.s006.docx]

## **Supplementary table 6: frequency of glucose tests by waist circumference categories at the national level**

| **Year** | **Sex** | **Normal waist circumference** | **Normal waist circumference lower limit** | **Normal waist circumference upper limit** | **Central obesity** | **Central obesity lower limit** | **Central obesity upper limit** |
| --- | --- | --- | --- | --- | --- | --- | --- |
| 2018 | Men | 0.1756 | 0.1543 | 0.1992 | 0.8244 | 0.8008 | 0.8457 |
| 2019 | Men | 0.1621 | 0.1407 | 0.1861 | 0.8379 | 0.8139 | 0.8593 |
| 2018 | Women | 0.0678 | 0.0527 | 0.0867 | 0.9322 | 0.9133 | 0.9473 |
| 2019 | Women | 0.0496 | 0.0390 | 0.0630 | 0.9504 | 0.9370 | 0.9610 |

Results are presents as proportions. Multiply these by 100 to get percentages.
