# Supplementary material for: Who is getting screened for diabetes according to body mass index and waist circumference categories in Peru? a pooled analysis of national surveys between 2015 and 2019
Source: PLoS One. 2021 Aug 27;16(8):e0256809. doi: 10.1371/journal.pone.0256809 (PMC8396776; doi:10.1371/journal.pone.0256809)
Supplement: S7 Table — (DOCX) [file pone.0256809.s007.docx]

## **Supplementary table 7: frequency of glucose tests by body mass index categories at the sub-national level**

| **Year** | **Region** | **Sex** | **Normal weight** | **Normal weight lower limit** | **Normal weight upper limit** | **Overweight** | **Overweight lower limit** | **Overweight upper limit** | **Obesity** | **Obesity lower limit** | **Obesity upper limit** |
| --- | --- | --- | --- | --- | --- | --- | --- | --- | --- | --- | --- |
| 2015 | Amazonas | Men | 0.3714 | 0.2061 | 0.5735 | 0.4938 | 0.3132 | 0.6760 | 0.1348 | 0.0569 | 0.2870 |
| 2016 | Amazonas | Men | 0.3526 | 0.2052 | 0.5346 | 0.5462 | 0.3867 | 0.6967 | 0.1012 | 0.0424 | 0.2226 |
| 2017 | Amazonas | Men | 0.3503 | 0.2035 | 0.5323 | 0.4845 | 0.3386 | 0.6330 | 0.1652 | 0.0722 | 0.3350 |
| 2018 | Amazonas | Men | 0.2941 | 0.1783 | 0.4444 | 0.4135 | 0.2725 | 0.5702 | 0.2925 | 0.1705 | 0.4540 |
| 2019 | Amazonas | Men | 0.3503 | 0.2401 | 0.4792 | 0.3449 | 0.2224 | 0.4922 | 0.3047 | 0.1988 | 0.4364 |
| 2015 | Ancash | Men | 0.2521 | 0.1596 | 0.3743 | 0.4543 | 0.3178 | 0.5981 | 0.2936 | 0.1935 | 0.4186 |
| 2016 | Ancash | Men | 0.1946 | 0.0809 | 0.3986 | 0.5111 | 0.3421 | 0.6776 | 0.2944 | 0.1714 | 0.4569 |
| 2017 | Ancash | Men | 0.2616 | 0.1331 | 0.4497 | 0.5372 | 0.3710 | 0.6956 | 0.2012 | 0.1026 | 0.3568 |
| 2018 | Ancash | Men | 0.0935 | 0.0392 | 0.2067 | 0.5344 | 0.3744 | 0.6877 | 0.3721 | 0.2340 | 0.5348 |
| 2019 | Ancash | Men | 0.1002 | 0.0410 | 0.2248 | 0.5012 | 0.3507 | 0.6515 | 0.3986 | 0.2527 | 0.5649 |
| 2015 | Apurimac | Men | 0.3699 | 0.2157 | 0.5563 | 0.4912 | 0.2892 | 0.6961 | 0.1389 | 0.0457 | 0.3520 |
| 2016 | Apurimac | Men | 0.2956 | 0.1702 | 0.4619 | 0.6016 | 0.4349 | 0.7477 | 0.1028 | 0.0435 | 0.2238 |
| 2017 | Apurimac | Men | 0.4596 | 0.3192 | 0.6069 | 0.4055 | 0.2774 | 0.5478 | 0.1349 | 0.0609 | 0.2728 |
| 2018 | Apurimac | Men | 0.4881 | 0.3473 | 0.6309 | 0.3957 | 0.2723 | 0.5339 | 0.1162 | 0.0524 | 0.2381 |
| 2019 | Apurimac | Men | 0.4611 | 0.3340 | 0.5934 | 0.4498 | 0.3279 | 0.5779 | 0.0892 | 0.0404 | 0.1856 |
| 2015 | Arequipa | Men | 0.1746 | 0.0925 | 0.3049 | 0.4636 | 0.3371 | 0.5950 | 0.3619 | 0.2476 | 0.4942 |
| 2016 | Arequipa | Men | 0.1776 | 0.1109 | 0.2721 | 0.4791 | 0.3732 | 0.5869 | 0.3433 | 0.2481 | 0.4531 |
| 2017 | Arequipa | Men | 0.1568 | 0.0936 | 0.2510 | 0.6152 | 0.5030 | 0.7163 | 0.2280 | 0.1460 | 0.3378 |
| 2018 | Arequipa | Men | 0.2378 | 0.1605 | 0.3373 | 0.5020 | 0.3859 | 0.6180 | 0.2602 | 0.1834 | 0.3552 |
| 2019 | Arequipa | Men | 0.0915 | 0.0404 | 0.1941 | 0.5722 | 0.4411 | 0.6938 | 0.3364 | 0.2246 | 0.4700 |
| 2015 | Ayacucho | Men | 0.3270 | 0.1983 | 0.4885 | 0.5009 | 0.3565 | 0.6453 | 0.1720 | 0.0899 | 0.3040 |
| 2016 | Ayacucho | Men | 0.2817 | 0.1636 | 0.4403 | 0.5396 | 0.3668 | 0.7034 | 0.1787 | 0.0905 | 0.3224 |
| 2017 | Ayacucho | Men | 0.4300 | 0.3018 | 0.5684 | 0.4855 | 0.3633 | 0.6094 | 0.0845 | 0.0399 | 0.1700 |
| 2018 | Ayacucho | Men | 0.3008 | 0.1986 | 0.4276 | 0.5823 | 0.4543 | 0.7001 | 0.1169 | 0.0667 | 0.1969 |
| 2019 | Ayacucho | Men | 0.2571 | 0.1608 | 0.3845 | 0.5619 | 0.4312 | 0.6845 | 0.1811 | 0.1059 | 0.2923 |
| 2015 | Cajamarca | Men | 0.2591 | 0.1353 | 0.4387 | 0.4968 | 0.2858 | 0.7089 | 0.2441 | 0.1141 | 0.4473 |
| 2016 | Cajamarca | Men | 0.2704 | 0.1010 | 0.5500 | 0.6831 | 0.4495 | 0.8505 | 0.0465 | 0.0075 | 0.2390 |
| 2017 | Cajamarca | Men | 0.2079 | 0.0914 | 0.4066 | 0.2743 | 0.1376 | 0.4725 | 0.5178 | 0.2914 | 0.7371 |
| 2018 | Cajamarca | Men | 0.3569 | 0.1730 | 0.5955 | 0.3308 | 0.1414 | 0.5974 | 0.3123 | 0.1424 | 0.5538 |
| 2019 | Cajamarca | Men | 0.4740 | 0.3201 | 0.6330 | 0.3679 | 0.2068 | 0.5651 | 0.1581 | 0.0757 | 0.3011 |
| 2015 | Callao | Men | 0.2043 | 0.1197 | 0.3266 | 0.4861 | 0.3731 | 0.6006 | 0.3096 | 0.2207 | 0.4152 |
| 2016 | Callao | Men | 0.1231 | 0.0680 | 0.2127 | 0.5152 | 0.3992 | 0.6297 | 0.3616 | 0.2701 | 0.4644 |
| 2017 | Callao | Men | 0.1728 | 0.1087 | 0.2634 | 0.5704 | 0.4692 | 0.6660 | 0.2568 | 0.1779 | 0.3557 |
| 2018 | Callao | Men | 0.1436 | 0.0887 | 0.2243 | 0.5293 | 0.4406 | 0.6163 | 0.3270 | 0.2440 | 0.4225 |
| 2019 | Callao | Men | 0.2179 | 0.1425 | 0.3185 | 0.4990 | 0.3826 | 0.6156 | 0.2830 | 0.1944 | 0.3923 |
| 2015 | Cusco | Men | 0.3154 | 0.1724 | 0.5047 | 0.5428 | 0.3554 | 0.7189 | 0.1418 | 0.0660 | 0.2787 |
| 2016 | Cusco | Men | 0.3782 | 0.2272 | 0.5573 | 0.3749 | 0.2222 | 0.5574 | 0.2468 | 0.1163 | 0.4494 |
| 2017 | Cusco | Men | 0.3061 | 0.1966 | 0.4430 | 0.4475 | 0.3130 | 0.5903 | 0.2463 | 0.1342 | 0.4080 |
| 2018 | Cusco | Men | 0.3200 | 0.1718 | 0.5163 | 0.4416 | 0.2693 | 0.6294 | 0.2384 | 0.1141 | 0.4319 |
| 2019 | Cusco | Men | 0.4068 | 0.2606 | 0.5715 | 0.4476 | 0.2981 | 0.6072 | 0.1457 | 0.0774 | 0.2575 |
| 2015 | Huancavelica | Men | 0.4291 | 0.2485 | 0.6308 | 0.4627 | 0.2729 | 0.6640 | 0.1082 | 0.0319 | 0.3085 |
| 2016 | Huancavelica | Men | 0.5983 | 0.3912 | 0.7753 | 0.3680 | 0.1951 | 0.5831 | 0.0337 | 0.0073 | 0.1413 |
| 2017 | Huancavelica | Men | 0.4158 | 0.2509 | 0.6020 | 0.4451 | 0.2838 | 0.6189 | 0.1391 | 0.0520 | 0.3224 |
| 2018 | Huancavelica | Men | 0.5254 | 0.3429 | 0.7014 | 0.3385 | 0.2226 | 0.4777 | 0.1361 | 0.0624 | 0.2715 |
| 2019 | Huancavelica | Men | 0.4797 | 0.3095 | 0.6548 | 0.3967 | 0.2375 | 0.5813 | 0.1236 | 0.0515 | 0.2678 |
| 2015 | Huanuco | Men | 0.3484 | 0.1791 | 0.5671 | 0.4259 | 0.2285 | 0.6502 | 0.2257 | 0.0998 | 0.4339 |
| 2016 | Huanuco | Men | 0.4792 | 0.3090 | 0.6543 | 0.3363 | 0.1988 | 0.5087 | 0.1845 | 0.0846 | 0.3564 |
| 2017 | Huanuco | Men | 0.3772 | 0.2393 | 0.5384 | 0.3683 | 0.2423 | 0.5153 | 0.2545 | 0.1367 | 0.4240 |
| 2018 | Huanuco | Men | 0.2266 | 0.1378 | 0.3495 | 0.5669 | 0.4104 | 0.7111 | 0.2065 | 0.1130 | 0.3471 |
| 2019 | Huanuco | Men | 0.2639 | 0.1595 | 0.4038 | 0.3926 | 0.2531 | 0.5522 | 0.3435 | 0.2089 | 0.5089 |
| 2015 | Ica | Men | 0.2389 | 0.1416 | 0.3741 | 0.4211 | 0.3053 | 0.5463 | 0.3399 | 0.2309 | 0.4690 |
| 2016 | Ica | Men | 0.1691 | 0.0883 | 0.2996 | 0.4311 | 0.3129 | 0.5578 | 0.3998 | 0.2863 | 0.5252 |
| 2017 | Ica | Men | 0.1918 | 0.1126 | 0.3073 | 0.4474 | 0.3497 | 0.5493 | 0.3608 | 0.2640 | 0.4705 |
| 2018 | Ica | Men | 0.1546 | 0.0926 | 0.2467 | 0.3829 | 0.2856 | 0.4907 | 0.4625 | 0.3544 | 0.5743 |
| 2019 | Ica | Men | 0.1560 | 0.0953 | 0.2450 | 0.3304 | 0.2423 | 0.4322 | 0.5136 | 0.4014 | 0.6245 |
| 2015 | Junin | Men | 0.2170 | 0.1155 | 0.3703 | 0.6632 | 0.4990 | 0.7956 | 0.1198 | 0.0516 | 0.2540 |
| 2016 | Junin | Men | 0.2724 | 0.1611 | 0.4220 | 0.5318 | 0.3581 | 0.6982 | 0.1958 | 0.0959 | 0.3585 |
| 2017 | Junin | Men | 0.2923 | 0.1658 | 0.4620 | 0.4401 | 0.2728 | 0.6222 | 0.2676 | 0.1433 | 0.4438 |
| 2018 | Junin | Men | 0.3276 | 0.1896 | 0.5037 | 0.3776 | 0.2414 | 0.5363 | 0.2948 | 0.1722 | 0.4566 |
| 2019 | Junin | Men | 0.2387 | 0.1298 | 0.3975 | 0.6007 | 0.4366 | 0.7450 | 0.1605 | 0.0750 | 0.3107 |
| 2015 | La Libertad | Men | 0.3720 | 0.2305 | 0.5396 | 0.3315 | 0.2300 | 0.4516 | 0.2965 | 0.1928 | 0.4264 |
| 2016 | La Libertad | Men | 0.1810 | 0.0876 | 0.3372 | 0.5200 | 0.3962 | 0.6413 | 0.2990 | 0.2001 | 0.4210 |
| 2017 | La Libertad | Men | 0.1217 | 0.0587 | 0.2352 | 0.6578 | 0.5339 | 0.7634 | 0.2205 | 0.1369 | 0.3353 |
| 2018 | La Libertad | Men | 0.1973 | 0.1103 | 0.3276 | 0.4842 | 0.3409 | 0.6302 | 0.3185 | 0.1976 | 0.4701 |
| 2019 | La Libertad | Men | 0.2516 | 0.1496 | 0.3913 | 0.5220 | 0.3665 | 0.6733 | 0.2264 | 0.1183 | 0.3895 |
| 2015 | Lambayeque | Men | 0.1717 | 0.0984 | 0.2827 | 0.4701 | 0.3340 | 0.6108 | 0.3581 | 0.2344 | 0.5042 |
| 2016 | Lambayeque | Men | 0.1691 | 0.0837 | 0.3120 | 0.4356 | 0.3090 | 0.5713 | 0.3953 | 0.2586 | 0.5506 |
| 2017 | Lambayeque | Men | 0.1979 | 0.1208 | 0.3072 | 0.4622 | 0.3337 | 0.5959 | 0.3399 | 0.2257 | 0.4762 |
| 2018 | Lambayeque | Men | 0.1764 | 0.1008 | 0.2903 | 0.4744 | 0.3584 | 0.5932 | 0.3492 | 0.2579 | 0.4532 |
| 2019 | Lambayeque | Men | 0.2481 | 0.1487 | 0.3839 | 0.4898 | 0.3667 | 0.6142 | 0.2621 | 0.1717 | 0.3784 |
| 2015 | Lima | Men | 0.1482 | 0.1048 | 0.2054 | 0.4971 | 0.4258 | 0.5685 | 0.3547 | 0.2906 | 0.4245 |
| 2016 | Lima | Men | 0.1772 | 0.1303 | 0.2365 | 0.5045 | 0.4349 | 0.5739 | 0.3183 | 0.2557 | 0.3882 |
| 2017 | Lima | Men | 0.1447 | 0.1038 | 0.1983 | 0.5202 | 0.4557 | 0.5840 | 0.3351 | 0.2709 | 0.4059 |
| 2018 | Lima | Men | 0.1670 | 0.1229 | 0.2229 | 0.4541 | 0.3864 | 0.5237 | 0.3788 | 0.3164 | 0.4455 |
| 2019 | Lima | Men | 0.1238 | 0.0894 | 0.1688 | 0.4794 | 0.4166 | 0.5429 | 0.3969 | 0.3394 | 0.4573 |
| 2015 | Loreto | Men | 0.2817 | 0.1679 | 0.4325 | 0.5780 | 0.4535 | 0.6933 | 0.1403 | 0.0693 | 0.2634 |
| 2016 | Loreto | Men | 0.2534 | 0.1563 | 0.3834 | 0.4252 | 0.2964 | 0.5650 | 0.3215 | 0.2068 | 0.4625 |
| 2017 | Loreto | Men | 0.3185 | 0.2097 | 0.4514 | 0.3995 | 0.2817 | 0.5302 | 0.2820 | 0.1888 | 0.3986 |
| 2018 | Loreto | Men | 0.2197 | 0.1342 | 0.3384 | 0.5255 | 0.3975 | 0.6502 | 0.2548 | 0.1567 | 0.3862 |
| 2019 | Loreto | Men | 0.2855 | 0.1768 | 0.4264 | 0.3883 | 0.2583 | 0.5364 | 0.3262 | 0.2140 | 0.4626 |
| 2015 | Madre de Dios | Men | 0.1132 | 0.0492 | 0.2395 | 0.4870 | 0.3469 | 0.6292 | 0.3998 | 0.2563 | 0.5629 |
| 2016 | Madre de Dios | Men | 0.1887 | 0.0988 | 0.3304 | 0.3335 | 0.2221 | 0.4672 | 0.4778 | 0.3475 | 0.6111 |
| 2017 | Madre de Dios | Men | 0.1561 | 0.0730 | 0.3027 | 0.4159 | 0.2913 | 0.5523 | 0.4280 | 0.2905 | 0.5777 |
| 2018 | Madre de Dios | Men | 0.1566 | 0.0835 | 0.2743 | 0.4487 | 0.3119 | 0.5936 | 0.3948 | 0.2610 | 0.5464 |
| 2019 | Madre de Dios | Men | 0.1663 | 0.0907 | 0.2852 | 0.4544 | 0.3470 | 0.5662 | 0.3793 | 0.2779 | 0.4924 |
| 2015 | Moquegua | Men | 0.1100 | 0.0611 | 0.1901 | 0.5675 | 0.4516 | 0.6765 | 0.3225 | 0.2346 | 0.4249 |
| 2016 | Moquegua | Men | 0.2165 | 0.1240 | 0.3505 | 0.5273 | 0.4101 | 0.6415 | 0.2562 | 0.1734 | 0.3612 |
| 2017 | Moquegua | Men | 0.1342 | 0.0792 | 0.2183 | 0.3874 | 0.2937 | 0.4902 | 0.4784 | 0.3658 | 0.5932 |
| 2018 | Moquegua | Men | 0.1833 | 0.1230 | 0.2642 | 0.3956 | 0.3007 | 0.4990 | 0.4212 | 0.3250 | 0.5237 |
| 2019 | Moquegua | Men | 0.1560 | 0.0966 | 0.2421 | 0.4580 | 0.3594 | 0.5600 | 0.3860 | 0.2992 | 0.4807 |
| 2015 | Pasco | Men | 0.3225 | 0.1923 | 0.4878 | 0.6054 | 0.4472 | 0.7442 | 0.0720 | 0.0256 | 0.1863 |
| 2016 | Pasco | Men | 0.3853 | 0.2489 | 0.5424 | 0.4926 | 0.3394 | 0.6472 | 0.1222 | 0.0498 | 0.2700 |
| 2017 | Pasco | Men | 0.3948 | 0.2731 | 0.5311 | 0.5306 | 0.3855 | 0.6708 | 0.0745 | 0.0310 | 0.1683 |
| 2018 | Pasco | Men | 0.2417 | 0.1419 | 0.3807 | 0.4782 | 0.3018 | 0.6602 | 0.2800 | 0.1114 | 0.5469 |
| 2019 | Pasco | Men | 0.3073 | 0.1953 | 0.4478 | 0.5338 | 0.3986 | 0.6642 | 0.1590 | 0.0897 | 0.2660 |
| 2015 | Piura | Men | 0.1555 | 0.0836 | 0.2708 | 0.5128 | 0.3693 | 0.6542 | 0.3317 | 0.2030 | 0.4916 |
| 2016 | Piura | Men | 0.1604 | 0.0806 | 0.2939 | 0.4891 | 0.3365 | 0.6438 | 0.3505 | 0.2125 | 0.5190 |
| 2017 | Piura | Men | 0.2428 | 0.1500 | 0.3680 | 0.5278 | 0.3822 | 0.6688 | 0.2294 | 0.1319 | 0.3685 |
| 2018 | Piura | Men | 0.1673 | 0.0904 | 0.2887 | 0.5101 | 0.4104 | 0.6089 | 0.3226 | 0.2215 | 0.4437 |
| 2019 | Piura | Men | 0.1403 | 0.0657 | 0.2747 | 0.5150 | 0.3707 | 0.6568 | 0.3448 | 0.2099 | 0.5104 |
| 2015 | Puno | Men | 0.4515 | 0.2383 | 0.6840 | 0.3799 | 0.1852 | 0.6228 | 0.1687 | 0.0524 | 0.4270 |
| 2016 | Puno | Men | 0.5717 | 0.3474 | 0.7699 | 0.3160 | 0.1358 | 0.5760 | 0.1123 | 0.0496 | 0.2346 |
| 2017 | Puno | Men | 0.3606 | 0.1651 | 0.6165 | 0.4295 | 0.2391 | 0.6433 | 0.2100 | 0.0823 | 0.4407 |
| 2018 | Puno | Men | 0.4210 | 0.2618 | 0.5984 | 0.4751 | 0.3296 | 0.6248 | 0.1040 | 0.0369 | 0.2599 |
| 2019 | Puno | Men | 0.2595 | 0.1345 | 0.4415 | 0.5253 | 0.3610 | 0.6844 | 0.2152 | 0.0979 | 0.4092 |
| 2015 | San Martin | Men | 0.3138 | 0.1906 | 0.4703 | 0.5135 | 0.3699 | 0.6549 | 0.1727 | 0.1010 | 0.2796 |
| 2016 | San Martin | Men | 0.3204 | 0.1923 | 0.4829 | 0.5414 | 0.3923 | 0.6835 | 0.1382 | 0.0701 | 0.2543 |
| 2017 | San Martin | Men | 0.2843 | 0.1872 | 0.4066 | 0.4937 | 0.3700 | 0.6182 | 0.2220 | 0.1382 | 0.3368 |
| 2018 | San Martin | Men | 0.2995 | 0.1978 | 0.4258 | 0.4647 | 0.3517 | 0.5814 | 0.2358 | 0.1513 | 0.3481 |
| 2019 | San Martin | Men | 0.1964 | 0.1250 | 0.2949 | 0.5127 | 0.4061 | 0.6182 | 0.2909 | 0.2022 | 0.3991 |
| 2015 | Tacna | Men | 0.2494 | 0.1405 | 0.4032 | 0.3386 | 0.2355 | 0.4598 | 0.4119 | 0.2876 | 0.5486 |
| 2016 | Tacna | Men | 0.1282 | 0.0672 | 0.2309 | 0.4953 | 0.3601 | 0.6312 | 0.3765 | 0.2528 | 0.5188 |
| 2017 | Tacna | Men | 0.1075 | 0.0597 | 0.1860 | 0.4296 | 0.3497 | 0.5134 | 0.4629 | 0.3730 | 0.5553 |
| 2018 | Tacna | Men | 0.1649 | 0.0933 | 0.2749 | 0.3434 | 0.2394 | 0.4649 | 0.4917 | 0.3689 | 0.6156 |
| 2019 | Tacna | Men | 0.1182 | 0.0644 | 0.2071 | 0.4902 | 0.3897 | 0.5914 | 0.3916 | 0.2915 | 0.5017 |
| 2015 | Tumbes | Men | 0.2042 | 0.1149 | 0.3363 | 0.3078 | 0.2134 | 0.4217 | 0.4880 | 0.3749 | 0.6024 |
| 2016 | Tumbes | Men | 0.1801 | 0.1062 | 0.2887 | 0.4789 | 0.3654 | 0.5946 | 0.3411 | 0.2242 | 0.4810 |
| 2017 | Tumbes | Men | 0.1877 | 0.1046 | 0.3139 | 0.4913 | 0.3660 | 0.6176 | 0.3210 | 0.2045 | 0.4649 |
| 2018 | Tumbes | Men | 0.2323 | 0.1405 | 0.3591 | 0.4735 | 0.3688 | 0.5807 | 0.2941 | 0.2101 | 0.3950 |
| 2019 | Tumbes | Men | 0.1914 | 0.1158 | 0.2995 | 0.5054 | 0.3927 | 0.6175 | 0.3033 | 0.2059 | 0.4223 |
| 2015 | Ucayali | Men | 0.2388 | 0.1281 | 0.4013 | 0.4351 | 0.2972 | 0.5838 | 0.3260 | 0.2033 | 0.4784 |
| 2016 | Ucayali | Men | 0.2758 | 0.1435 | 0.4639 | 0.3117 | 0.2022 | 0.4471 | 0.4126 | 0.2797 | 0.5596 |
| 2017 | Ucayali | Men | 0.3383 | 0.1868 | 0.5321 | 0.4361 | 0.2611 | 0.6287 | 0.2256 | 0.0976 | 0.4397 |
| 2018 | Ucayali | Men | 0.2477 | 0.1244 | 0.4328 | 0.4696 | 0.2812 | 0.6671 | 0.2827 | 0.1556 | 0.4573 |
| 2019 | Ucayali | Men | 0.1868 | 0.1056 | 0.3090 | 0.4306 | 0.2973 | 0.5748 | 0.3826 | 0.2578 | 0.5251 |
| 2015 | Amazonas | Women | 0.1148 | 0.0478 | 0.2511 | 0.4936 | 0.3559 | 0.6323 | 0.3916 | 0.2511 | 0.5526 |
| 2016 | Amazonas | Women | 0.1970 | 0.1114 | 0.3245 | 0.5416 | 0.4133 | 0.6647 | 0.2614 | 0.1728 | 0.3747 |
| 2017 | Amazonas | Women | 0.2529 | 0.1674 | 0.3630 | 0.4236 | 0.3196 | 0.5349 | 0.3234 | 0.2238 | 0.4421 |
| 2018 | Amazonas | Women | 0.2268 | 0.1444 | 0.3376 | 0.4935 | 0.3671 | 0.6208 | 0.2796 | 0.1814 | 0.4048 |
| 2019 | Amazonas | Women | 0.2111 | 0.1238 | 0.3362 | 0.5006 | 0.3904 | 0.6108 | 0.2883 | 0.2010 | 0.3947 |
| 2015 | Ancash | Women | 0.1228 | 0.0679 | 0.2120 | 0.4254 | 0.3214 | 0.5363 | 0.4519 | 0.3423 | 0.5663 |
| 2016 | Ancash | Women | 0.1993 | 0.1179 | 0.3167 | 0.3986 | 0.2556 | 0.5611 | 0.4022 | 0.2745 | 0.5447 |
| 2017 | Ancash | Women | 0.2121 | 0.1319 | 0.3230 | 0.3590 | 0.2520 | 0.4822 | 0.4289 | 0.3025 | 0.5652 |
| 2018 | Ancash | Women | 0.1497 | 0.0795 | 0.2641 | 0.4948 | 0.3889 | 0.6011 | 0.3556 | 0.2569 | 0.4682 |
| 2019 | Ancash | Women | 0.2347 | 0.1648 | 0.3230 | 0.3790 | 0.2898 | 0.4772 | 0.3862 | 0.2864 | 0.4966 |
| 2015 | Apurimac | Women | 0.3389 | 0.1979 | 0.5158 | 0.4000 | 0.2597 | 0.5589 | 0.2611 | 0.1559 | 0.4033 |
| 2016 | Apurimac | Women | 0.2375 | 0.1542 | 0.3473 | 0.3422 | 0.2385 | 0.4636 | 0.4203 | 0.3080 | 0.5414 |
| 2017 | Apurimac | Women | 0.3007 | 0.2165 | 0.4009 | 0.3425 | 0.2495 | 0.4493 | 0.3568 | 0.2581 | 0.4693 |
| 2018 | Apurimac | Women | 0.3303 | 0.2360 | 0.4405 | 0.4723 | 0.3613 | 0.5861 | 0.1974 | 0.1291 | 0.2899 |
| 2019 | Apurimac | Women | 0.2894 | 0.2077 | 0.3876 | 0.4595 | 0.3644 | 0.5577 | 0.2511 | 0.1719 | 0.3513 |
| 2015 | Arequipa | Women | 0.1411 | 0.0831 | 0.2294 | 0.4670 | 0.3630 | 0.5739 | 0.3919 | 0.2874 | 0.5074 |
| 2016 | Arequipa | Women | 0.1536 | 0.0870 | 0.2569 | 0.4057 | 0.2966 | 0.5249 | 0.4407 | 0.3387 | 0.5480 |
| 2017 | Arequipa | Women | 0.1940 | 0.1246 | 0.2893 | 0.3852 | 0.2909 | 0.4891 | 0.4207 | 0.3208 | 0.5276 |
| 2018 | Arequipa | Women | 0.1269 | 0.0767 | 0.2029 | 0.3941 | 0.2955 | 0.5022 | 0.4790 | 0.3832 | 0.5763 |
| 2019 | Arequipa | Women | 0.1844 | 0.1224 | 0.2681 | 0.3836 | 0.2735 | 0.5070 | 0.4321 | 0.3284 | 0.5420 |
| 2015 | Ayacucho | Women | 0.2469 | 0.1506 | 0.3774 | 0.4178 | 0.3096 | 0.5345 | 0.3353 | 0.2161 | 0.4800 |
| 2016 | Ayacucho | Women | 0.2613 | 0.1651 | 0.3875 | 0.3638 | 0.2597 | 0.4824 | 0.3749 | 0.2643 | 0.5004 |
| 2017 | Ayacucho | Women | 0.2571 | 0.1749 | 0.3610 | 0.5118 | 0.4140 | 0.6087 | 0.2311 | 0.1580 | 0.3249 |
| 2018 | Ayacucho | Women | 0.2231 | 0.1559 | 0.3087 | 0.4530 | 0.3595 | 0.5499 | 0.3239 | 0.2366 | 0.4255 |
| 2019 | Ayacucho | Women | 0.1746 | 0.1188 | 0.2493 | 0.5880 | 0.4885 | 0.6808 | 0.2373 | 0.1673 | 0.3252 |
| 2015 | Cajamarca | Women | 0.3320 | 0.2004 | 0.4965 | 0.3933 | 0.2464 | 0.5624 | 0.2747 | 0.1434 | 0.4614 |
| 2016 | Cajamarca | Women | 0.2603 | 0.1391 | 0.4337 | 0.5010 | 0.3199 | 0.6818 | 0.2388 | 0.0982 | 0.4747 |
| 2017 | Cajamarca | Women | 0.3362 | 0.2301 | 0.4618 | 0.3376 | 0.2322 | 0.4620 | 0.3262 | 0.2083 | 0.4712 |
| 2018 | Cajamarca | Women | 0.2652 | 0.1694 | 0.3898 | 0.4223 | 0.2820 | 0.5763 | 0.3125 | 0.2138 | 0.4318 |
| 2019 | Cajamarca | Women | 0.3271 | 0.2115 | 0.4683 | 0.4472 | 0.3088 | 0.5942 | 0.2258 | 0.1384 | 0.3460 |
| 2015 | Callao | Women | 0.1054 | 0.0622 | 0.1730 | 0.3958 | 0.2952 | 0.5062 | 0.4988 | 0.3990 | 0.5986 |
| 2016 | Callao | Women | 0.1162 | 0.0766 | 0.1724 | 0.4921 | 0.4081 | 0.5766 | 0.3917 | 0.3101 | 0.4798 |
| 2017 | Callao | Women | 0.1654 | 0.1063 | 0.2483 | 0.3560 | 0.2738 | 0.4476 | 0.4786 | 0.3914 | 0.5672 |
| 2018 | Callao | Women | 0.1463 | 0.0977 | 0.2135 | 0.3781 | 0.3025 | 0.4602 | 0.4755 | 0.3984 | 0.5538 |
| 2019 | Callao | Women | 0.1560 | 0.1020 | 0.2313 | 0.3943 | 0.3089 | 0.4866 | 0.4497 | 0.3647 | 0.5378 |
| 2015 | Cusco | Women | 0.3229 | 0.1943 | 0.4852 | 0.4173 | 0.2797 | 0.5692 | 0.2598 | 0.1467 | 0.4174 |
| 2016 | Cusco | Women | 0.1546 | 0.0636 | 0.3298 | 0.2715 | 0.1525 | 0.4356 | 0.5739 | 0.4151 | 0.7187 |
| 2017 | Cusco | Women | 0.2122 | 0.1228 | 0.3414 | 0.4370 | 0.3102 | 0.5726 | 0.3508 | 0.2420 | 0.4778 |
| 2018 | Cusco | Women | 0.1860 | 0.1093 | 0.2985 | 0.3555 | 0.2569 | 0.4681 | 0.4585 | 0.3584 | 0.5621 |
| 2019 | Cusco | Women | 0.2365 | 0.1405 | 0.3698 | 0.3619 | 0.2619 | 0.4755 | 0.4015 | 0.2935 | 0.5201 |
| 2015 | Huancavelica | Women | 0.2648 | 0.1479 | 0.4276 | 0.3100 | 0.1768 | 0.4846 | 0.4252 | 0.2524 | 0.6185 |
| 2016 | Huancavelica | Women | 0.3908 | 0.2503 | 0.5521 | 0.3981 | 0.2714 | 0.5400 | 0.2111 | 0.1202 | 0.3441 |
| 2017 | Huancavelica | Women | 0.3525 | 0.2210 | 0.5109 | 0.4045 | 0.2823 | 0.5399 | 0.2429 | 0.1388 | 0.3899 |
| 2018 | Huancavelica | Women | 0.2881 | 0.1882 | 0.4141 | 0.4101 | 0.2986 | 0.5317 | 0.3017 | 0.2162 | 0.4036 |
| 2019 | Huancavelica | Women | 0.4100 | 0.2713 | 0.5645 | 0.3910 | 0.2568 | 0.5440 | 0.1991 | 0.1211 | 0.3097 |
| 2015 | Huanuco | Women | 0.1365 | 0.0733 | 0.2400 | 0.5162 | 0.3869 | 0.6434 | 0.3474 | 0.2401 | 0.4727 |
| 2016 | Huanuco | Women | 0.2925 | 0.1801 | 0.4376 | 0.3649 | 0.2591 | 0.4857 | 0.3426 | 0.2414 | 0.4605 |
| 2017 | Huanuco | Women | 0.3021 | 0.2048 | 0.4211 | 0.3843 | 0.2855 | 0.4937 | 0.3136 | 0.2062 | 0.4457 |
| 2018 | Huanuco | Women | 0.3532 | 0.2390 | 0.4870 | 0.4495 | 0.3409 | 0.5632 | 0.1973 | 0.1199 | 0.3073 |
| 2019 | Huanuco | Women | 0.1974 | 0.1224 | 0.3026 | 0.4499 | 0.3500 | 0.5539 | 0.3527 | 0.2691 | 0.4465 |
| 2015 | Ica | Women | 0.1486 | 0.0943 | 0.2263 | 0.3586 | 0.2813 | 0.4441 | 0.4928 | 0.4090 | 0.5770 |
| 2016 | Ica | Women | 0.1387 | 0.0825 | 0.2238 | 0.4066 | 0.2975 | 0.5258 | 0.4548 | 0.3356 | 0.5793 |
| 2017 | Ica | Women | 0.1352 | 0.0894 | 0.1993 | 0.4135 | 0.3469 | 0.4835 | 0.4513 | 0.3777 | 0.5271 |
| 2018 | Ica | Women | 0.1227 | 0.0745 | 0.1955 | 0.3947 | 0.3190 | 0.4758 | 0.4826 | 0.3947 | 0.5716 |
| 2019 | Ica | Women | 0.1166 | 0.0692 | 0.1897 | 0.4122 | 0.3289 | 0.5009 | 0.4712 | 0.3794 | 0.5651 |
| 2015 | Junin | Women | 0.2305 | 0.1442 | 0.3474 | 0.4788 | 0.3686 | 0.5912 | 0.2907 | 0.1929 | 0.4127 |
| 2016 | Junin | Women | 0.4610 | 0.3270 | 0.6009 | 0.2815 | 0.1766 | 0.4172 | 0.2575 | 0.1652 | 0.3780 |
| 2017 | Junin | Women | 0.1383 | 0.0780 | 0.2333 | 0.4412 | 0.3379 | 0.5498 | 0.4206 | 0.3209 | 0.5271 |
| 2018 | Junin | Women | 0.2133 | 0.1304 | 0.3291 | 0.4370 | 0.3300 | 0.5503 | 0.3496 | 0.2480 | 0.4671 |
| 2019 | Junin | Women | 0.1298 | 0.0742 | 0.2172 | 0.4844 | 0.3771 | 0.5931 | 0.3858 | 0.2842 | 0.4984 |
| 2015 | La Libertad | Women | 0.1087 | 0.0622 | 0.1833 | 0.5080 | 0.3939 | 0.6213 | 0.3833 | 0.2878 | 0.4887 |
| 2016 | La Libertad | Women | 0.1531 | 0.0820 | 0.2679 | 0.3654 | 0.2501 | 0.4986 | 0.4815 | 0.3484 | 0.6172 |
| 2017 | La Libertad | Women | 0.1989 | 0.1204 | 0.3107 | 0.4984 | 0.3765 | 0.6206 | 0.3026 | 0.2018 | 0.4268 |
| 2018 | La Libertad | Women | 0.1085 | 0.0555 | 0.2013 | 0.4413 | 0.3315 | 0.5572 | 0.4501 | 0.3442 | 0.5608 |
| 2019 | La Libertad | Women | 0.1322 | 0.0710 | 0.2328 | 0.4689 | 0.3767 | 0.5633 | 0.3989 | 0.2973 | 0.5100 |
| 2015 | Lambayeque | Women | 0.2177 | 0.1418 | 0.3191 | 0.3485 | 0.2410 | 0.4740 | 0.4338 | 0.3098 | 0.5668 |
| 2016 | Lambayeque | Women | 0.2070 | 0.1449 | 0.2867 | 0.3588 | 0.2642 | 0.4659 | 0.4342 | 0.3387 | 0.5348 |
| 2017 | Lambayeque | Women | 0.1767 | 0.1148 | 0.2620 | 0.4497 | 0.3571 | 0.5459 | 0.3736 | 0.2900 | 0.4655 |
| 2018 | Lambayeque | Women | 0.2351 | 0.1688 | 0.3176 | 0.4552 | 0.3654 | 0.5480 | 0.3097 | 0.2346 | 0.3964 |
| 2019 | Lambayeque | Women | 0.2241 | 0.1611 | 0.3029 | 0.3564 | 0.2790 | 0.4422 | 0.4194 | 0.3341 | 0.5099 |
| 2015 | Lima | Women | 0.1908 | 0.1459 | 0.2454 | 0.4015 | 0.3434 | 0.4625 | 0.4077 | 0.3461 | 0.4725 |
| 2016 | Lima | Women | 0.1558 | 0.1158 | 0.2064 | 0.4057 | 0.3441 | 0.4704 | 0.4385 | 0.3808 | 0.4979 |
| 2017 | Lima | Women | 0.1770 | 0.1349 | 0.2288 | 0.4451 | 0.3891 | 0.5026 | 0.3778 | 0.3216 | 0.4376 |
| 2018 | Lima | Women | 0.1834 | 0.1444 | 0.2302 | 0.3814 | 0.3280 | 0.4379 | 0.4351 | 0.3814 | 0.4904 |
| 2019 | Lima | Women | 0.1753 | 0.1317 | 0.2295 | 0.4696 | 0.4093 | 0.5308 | 0.3550 | 0.3010 | 0.4130 |
| 2015 | Loreto | Women | 0.2983 | 0.1952 | 0.4269 | 0.4960 | 0.3784 | 0.6141 | 0.2057 | 0.1323 | 0.3054 |
| 2016 | Loreto | Women | 0.2662 | 0.1804 | 0.3741 | 0.3562 | 0.2504 | 0.4783 | 0.3775 | 0.2773 | 0.4894 |
| 2017 | Loreto | Women | 0.1825 | 0.1091 | 0.2893 | 0.4038 | 0.2971 | 0.5204 | 0.4137 | 0.3063 | 0.5301 |
| 2018 | Loreto | Women | 0.2006 | 0.1244 | 0.3072 | 0.4591 | 0.3344 | 0.5891 | 0.3403 | 0.2499 | 0.4440 |
| 2019 | Loreto | Women | 0.1798 | 0.1080 | 0.2842 | 0.3797 | 0.2806 | 0.4899 | 0.4405 | 0.3351 | 0.5515 |
| 2015 | Madre de Dios | Women | 0.0622 | 0.0245 | 0.1490 | 0.4880 | 0.3779 | 0.5993 | 0.4498 | 0.3392 | 0.5657 |
| 2016 | Madre de Dios | Women | 0.1589 | 0.0870 | 0.2724 | 0.3296 | 0.2274 | 0.4510 | 0.5115 | 0.3774 | 0.6439 |
| 2017 | Madre de Dios | Women | 0.1660 | 0.0961 | 0.2715 | 0.3307 | 0.2209 | 0.4627 | 0.5033 | 0.3792 | 0.6270 |
| 2018 | Madre de Dios | Women | 0.1299 | 0.0715 | 0.2246 | 0.4328 | 0.3110 | 0.5633 | 0.4373 | 0.3219 | 0.5600 |
| 2019 | Madre de Dios | Women | 0.0952 | 0.0474 | 0.1821 | 0.4247 | 0.3002 | 0.5595 | 0.4801 | 0.3485 | 0.6145 |
| 2015 | Moquegua | Women | 0.1465 | 0.0904 | 0.2287 | 0.4627 | 0.3537 | 0.5754 | 0.3908 | 0.2941 | 0.4968 |
| 2016 | Moquegua | Women | 0.1187 | 0.0731 | 0.1871 | 0.4828 | 0.3839 | 0.5831 | 0.3985 | 0.3052 | 0.4998 |
| 2017 | Moquegua | Women | 0.1320 | 0.0785 | 0.2136 | 0.3294 | 0.2314 | 0.4450 | 0.5385 | 0.4237 | 0.6494 |
| 2018 | Moquegua | Women | 0.0899 | 0.0521 | 0.1509 | 0.3058 | 0.2244 | 0.4016 | 0.6042 | 0.5105 | 0.6909 |
| 2019 | Moquegua | Women | 0.0874 | 0.0488 | 0.1515 | 0.4203 | 0.3239 | 0.5232 | 0.4923 | 0.3952 | 0.5900 |
| 2015 | Pasco | Women | 0.1756 | 0.0857 | 0.3263 | 0.4020 | 0.2606 | 0.5618 | 0.4224 | 0.2757 | 0.5842 |
| 2016 | Pasco | Women | 0.2175 | 0.1199 | 0.3619 | 0.3707 | 0.2527 | 0.5064 | 0.4118 | 0.2781 | 0.5601 |
| 2017 | Pasco | Women | 0.2440 | 0.1504 | 0.3704 | 0.2994 | 0.1860 | 0.4443 | 0.4566 | 0.3235 | 0.5962 |
| 2018 | Pasco | Women | 0.2135 | 0.1201 | 0.3506 | 0.4236 | 0.2896 | 0.5698 | 0.3629 | 0.2336 | 0.5156 |
| 2019 | Pasco | Women | 0.1275 | 0.0618 | 0.2448 | 0.5281 | 0.3963 | 0.6562 | 0.3443 | 0.2353 | 0.4727 |
| 2015 | Piura | Women | 0.1278 | 0.0721 | 0.2163 | 0.4601 | 0.3369 | 0.5883 | 0.4122 | 0.2967 | 0.5382 |
| 2016 | Piura | Women | 0.2175 | 0.1401 | 0.3216 | 0.3263 | 0.2287 | 0.4416 | 0.4562 | 0.3600 | 0.5559 |
| 2017 | Piura | Women | 0.2594 | 0.1824 | 0.3549 | 0.4412 | 0.3372 | 0.5506 | 0.2994 | 0.2152 | 0.3997 |
| 2018 | Piura | Women | 0.1396 | 0.0775 | 0.2388 | 0.4183 | 0.3086 | 0.5368 | 0.4420 | 0.3409 | 0.5482 |
| 2019 | Piura | Women | 0.1458 | 0.0868 | 0.2347 | 0.4503 | 0.3465 | 0.5586 | 0.4039 | 0.3121 | 0.5029 |
| 2015 | Puno | Women | 0.2865 | 0.1276 | 0.5243 | 0.2718 | 0.1251 | 0.4935 | 0.4417 | 0.2643 | 0.6354 |
| 2016 | Puno | Women | 0.1788 | 0.0883 | 0.3286 | 0.4651 | 0.2969 | 0.6417 | 0.3561 | 0.1987 | 0.5522 |
| 2017 | Puno | Women | 0.2465 | 0.1327 | 0.4116 | 0.5454 | 0.4093 | 0.6750 | 0.2081 | 0.1131 | 0.3514 |
| 2018 | Puno | Women | 0.1614 | 0.0846 | 0.2863 | 0.4755 | 0.3576 | 0.5961 | 0.3631 | 0.2626 | 0.4772 |
| 2019 | Puno | Women | 0.2374 | 0.1319 | 0.3893 | 0.3333 | 0.2228 | 0.4657 | 0.4293 | 0.2999 | 0.5692 |
| 2015 | San Martin | Women | 0.2973 | 0.2067 | 0.4073 | 0.4288 | 0.3300 | 0.5337 | 0.2738 | 0.1843 | 0.3863 |
| 2016 | San Martin | Women | 0.2600 | 0.1793 | 0.3610 | 0.4774 | 0.3597 | 0.5977 | 0.2626 | 0.1846 | 0.3590 |
| 2017 | San Martin | Women | 0.2599 | 0.1759 | 0.3661 | 0.3415 | 0.2404 | 0.4594 | 0.3986 | 0.2972 | 0.5096 |
| 2018 | San Martin | Women | 0.1706 | 0.1033 | 0.2686 | 0.4248 | 0.3229 | 0.5337 | 0.4046 | 0.3079 | 0.5093 |
| 2019 | San Martin | Women | 0.2455 | 0.1570 | 0.3626 | 0.4166 | 0.3169 | 0.5237 | 0.3378 | 0.2599 | 0.4256 |
| 2015 | Tacna | Women | 0.1532 | 0.0880 | 0.2533 | 0.3832 | 0.2703 | 0.5103 | 0.4636 | 0.3398 | 0.5921 |
| 2016 | Tacna | Women | 0.1115 | 0.0598 | 0.1982 | 0.4595 | 0.3372 | 0.5868 | 0.4291 | 0.3289 | 0.5354 |
| 2017 | Tacna | Women | 0.1223 | 0.0722 | 0.1997 | 0.3182 | 0.1981 | 0.4686 | 0.5594 | 0.4121 | 0.6971 |
| 2018 | Tacna | Women | 0.1334 | 0.0825 | 0.2085 | 0.3654 | 0.2772 | 0.4638 | 0.5012 | 0.4054 | 0.5968 |
| 2019 | Tacna | Women | 0.1443 | 0.0899 | 0.2238 | 0.2613 | 0.1927 | 0.3439 | 0.5944 | 0.5056 | 0.6774 |
| 2015 | Tumbes | Women | 0.1372 | 0.0784 | 0.2293 | 0.4647 | 0.3546 | 0.5784 | 0.3980 | 0.2863 | 0.5215 |
| 2016 | Tumbes | Women | 0.1947 | 0.1273 | 0.2860 | 0.3389 | 0.2364 | 0.4592 | 0.4664 | 0.3509 | 0.5856 |
| 2017 | Tumbes | Women | 0.2415 | 0.1665 | 0.3365 | 0.3291 | 0.2447 | 0.4261 | 0.4295 | 0.3340 | 0.5305 |
| 2018 | Tumbes | Women | 0.1786 | 0.1103 | 0.2761 | 0.3660 | 0.2656 | 0.4796 | 0.4554 | 0.3536 | 0.5610 |
| 2019 | Tumbes | Women | 0.1782 | 0.1058 | 0.2843 | 0.4206 | 0.3228 | 0.5251 | 0.4012 | 0.2969 | 0.5153 |
| 2015 | Ucayali | Women | 0.1862 | 0.1154 | 0.2863 | 0.4684 | 0.3527 | 0.5876 | 0.3455 | 0.2375 | 0.4721 |
| 2016 | Ucayali | Women | 0.2670 | 0.1757 | 0.3836 | 0.4992 | 0.3813 | 0.6173 | 0.2338 | 0.1537 | 0.3389 |
| 2017 | Ucayali | Women | 0.2202 | 0.1400 | 0.3288 | 0.3960 | 0.2927 | 0.5095 | 0.3838 | 0.2707 | 0.5111 |
| 2018 | Ucayali | Women | 0.1173 | 0.0584 | 0.2216 | 0.4340 | 0.3208 | 0.5545 | 0.4487 | 0.3233 | 0.5810 |
| 2019 | Ucayali | Women | 0.1820 | 0.1114 | 0.2831 | 0.4425 | 0.3218 | 0.5705 | 0.3755 | 0.2614 | 0.5052 |

Results are presented as proportions. Multiply by 100 to have percentages.
