# Supplementary material for: Who is getting screened for diabetes according to body mass index and waist circumference categories in Peru? a pooled analysis of national surveys between 2015 and 2019
Source: PLoS One. 2021 Aug 27;16(8):e0256809. doi: 10.1371/journal.pone.0256809 (PMC8396776; doi:10.1371/journal.pone.0256809)
Supplement: S8 Table — (DOCX) [file pone.0256809.s008.docx]

## **Supplementary table 8: frequency of glucose tests by waist circumference categories at the sub-national level**

| **Year** | **Region** | **Sex** | **Normal waist circumference** | **Normal waist circumference lower limit** | **Normal waist circumference upper limit** | **Central obesity** | **Central obesity lower limit** | **Central obesity upper limit** |
| --- | --- | --- | --- | --- | --- | --- | --- | --- |
| 2018 | Amazonas | Men | 0.3261 | 0.2107 | 0.4673 | 0.6739 | 0.5327 | 0.7893 |
| 2019 | Amazonas | Men | 0.3239 | 0.2095 | 0.4640 | 0.6761 | 0.5360 | 0.7905 |
| 2018 | Ancash | Men | 0.0819 | 0.0315 | 0.1964 | 0.9181 | 0.8036 | 0.9685 |
| 2019 | Ancash | Men | 0.1571 | 0.0743 | 0.3021 | 0.8429 | 0.6979 | 0.9257 |
| 2018 | Apurimac | Men | 0.5870 | 0.4285 | 0.7293 | 0.4130 | 0.2707 | 0.5715 |
| 2019 | Apurimac | Men | 0.3995 | 0.2820 | 0.5297 | 0.6005 | 0.4703 | 0.7180 |
| 2018 | Arequipa | Men | 0.1456 | 0.0843 | 0.2397 | 0.8544 | 0.7603 | 0.9157 |
| 2019 | Arequipa | Men | 0.0819 | 0.0337 | 0.1856 | 0.9181 | 0.8144 | 0.9663 |
| 2018 | Ayacucho | Men | 0.2524 | 0.1626 | 0.3698 | 0.7476 | 0.6302 | 0.8374 |
| 2019 | Ayacucho | Men | 0.2663 | 0.1702 | 0.3909 | 0.7337 | 0.6091 | 0.8298 |
| 2018 | Cajamarca | Men | 0.4085 | 0.2085 | 0.6443 | 0.5915 | 0.3557 | 0.7915 |
| 2019 | Cajamarca | Men | 0.4388 | 0.2838 | 0.6068 | 0.5612 | 0.3932 | 0.7162 |
| 2018 | Callao | Men | 0.1296 | 0.0811 | 0.2008 | 0.8704 | 0.7992 | 0.9189 |
| 2019 | Callao | Men | 0.2009 | 0.1259 | 0.3050 | 0.7991 | 0.6950 | 0.8741 |
| 2018 | Cusco | Men | 0.3200 | 0.1718 | 0.5163 | 0.6800 | 0.4837 | 0.8282 |
| 2019 | Cusco | Men | 0.3452 | 0.2100 | 0.5112 | 0.6548 | 0.4888 | 0.7900 |
| 2018 | Huancavelica | Men | 0.5560 | 0.3617 | 0.7345 | 0.4440 | 0.2655 | 0.6383 |
| 2019 | Huancavelica | Men | 0.5385 | 0.3629 | 0.7050 | 0.4615 | 0.2950 | 0.6371 |
| 2018 | Huanuco | Men | 0.1834 | 0.1090 | 0.2921 | 0.8166 | 0.7079 | 0.8910 |
| 2019 | Huanuco | Men | 0.1945 | 0.1193 | 0.3010 | 0.8055 | 0.6990 | 0.8807 |
| 2018 | Ica | Men | 0.1250 | 0.0673 | 0.2206 | 0.8750 | 0.7794 | 0.9327 |
| 2019 | Ica | Men | 0.1532 | 0.0931 | 0.2417 | 0.8468 | 0.7583 | 0.9069 |
| 2018 | Junin | Men | 0.2965 | 0.1777 | 0.4511 | 0.7035 | 0.5489 | 0.8223 |
| 2019 | Junin | Men | 0.2789 | 0.1718 | 0.4189 | 0.7211 | 0.5811 | 0.8282 |
| 2018 | La Libertad | Men | 0.2082 | 0.1209 | 0.3347 | 0.7918 | 0.6653 | 0.8791 |
| 2019 | La Libertad | Men | 0.2141 | 0.1217 | 0.3488 | 0.7859 | 0.6512 | 0.8783 |
| 2018 | Lambayeque | Men | 0.1210 | 0.0676 | 0.2073 | 0.8790 | 0.7927 | 0.9324 |
| 2019 | Lambayeque | Men | 0.2225 | 0.1286 | 0.3570 | 0.7775 | 0.6430 | 0.8714 |
| 2018 | Lima | Men | 0.1331 | 0.0982 | 0.1781 | 0.8669 | 0.8219 | 0.9018 |
| 2019 | Lima | Men | 0.1069 | 0.0752 | 0.1497 | 0.8931 | 0.8503 | 0.9248 |
| 2018 | Loreto | Men | 0.2023 | 0.1188 | 0.3229 | 0.7977 | 0.6771 | 0.8812 |
| 2019 | Loreto | Men | 0.2952 | 0.1849 | 0.4361 | 0.7048 | 0.5639 | 0.8151 |
| 2018 | Madre de Dios | Men | 0.1249 | 0.0559 | 0.2559 | 0.8751 | 0.7441 | 0.9441 |
| 2019 | Madre de Dios | Men | 0.1588 | 0.0807 | 0.2888 | 0.8412 | 0.7112 | 0.9193 |
| 2018 | Moquegua | Men | 0.1206 | 0.0739 | 0.1908 | 0.8794 | 0.8092 | 0.9261 |
| 2019 | Moquegua | Men | 0.1117 | 0.0667 | 0.1813 | 0.8883 | 0.8187 | 0.9333 |
| 2018 | Pasco | Men | 0.2988 | 0.1669 | 0.4754 | 0.7012 | 0.5246 | 0.8331 |
| 2019 | Pasco | Men | 0.2634 | 0.1587 | 0.4041 | 0.7366 | 0.5959 | 0.8413 |
| 2018 | Piura | Men | 0.2288 | 0.1360 | 0.3586 | 0.7712 | 0.6414 | 0.8640 |
| 2019 | Piura | Men | 0.1559 | 0.0776 | 0.2888 | 0.8441 | 0.7112 | 0.9224 |
| 2018 | Puno | Men | 0.3568 | 0.2278 | 0.5105 | 0.6432 | 0.4895 | 0.7722 |
| 2019 | Puno | Men | 0.2484 | 0.1232 | 0.4372 | 0.7516 | 0.5628 | 0.8768 |
| 2018 | San Martin | Men | 0.2606 | 0.1659 | 0.3846 | 0.7394 | 0.6154 | 0.8341 |
| 2019 | San Martin | Men | 0.1491 | 0.0858 | 0.2465 | 0.8509 | 0.7535 | 0.9142 |
| 2018 | Tacna | Men | 0.0760 | 0.0307 | 0.1762 | 0.9240 | 0.8238 | 0.9693 |
| 2019 | Tacna | Men | 0.0767 | 0.0373 | 0.1513 | 0.9233 | 0.8487 | 0.9627 |
| 2018 | Tumbes | Men | 0.1434 | 0.0903 | 0.2202 | 0.8566 | 0.7798 | 0.9097 |
| 2019 | Tumbes | Men | 0.0852 | 0.0404 | 0.1710 | 0.9148 | 0.8290 | 0.9596 |
| 2018 | Ucayali | Men | 0.1537 | 0.0616 | 0.3345 | 0.8463 | 0.6655 | 0.9384 |
| 2019 | Ucayali | Men | 0.1676 | 0.0915 | 0.2870 | 0.8324 | 0.7130 | 0.9085 |
| 2018 | Amazonas | Women | 0.0798 | 0.0332 | 0.1795 | 0.9202 | 0.8205 | 0.9668 |
| 2019 | Amazonas | Women | 0.0873 | 0.0409 | 0.1768 | 0.9127 | 0.8232 | 0.9591 |
| 2018 | Ancash | Women | 0.0273 | 0.0100 | 0.0722 | 0.9727 | 0.9278 | 0.9900 |
| 2019 | Ancash | Women | 0.0320 | 0.0108 | 0.0912 | 0.9680 | 0.9088 | 0.9892 |
| 2018 | Apurimac | Women | 0.2655 | 0.1757 | 0.3801 | 0.7345 | 0.6199 | 0.8243 |
| 2019 | Apurimac | Women | 0.1835 | 0.1211 | 0.2683 | 0.8165 | 0.7317 | 0.8789 |
| 2018 | Arequipa | Women | 0.0489 | 0.0200 | 0.1150 | 0.9511 | 0.8850 | 0.9800 |
| 2019 | Arequipa | Women | 0.0641 | 0.0295 | 0.1339 | 0.9359 | 0.8661 | 0.9705 |
| 2018 | Ayacucho | Women | 0.0908 | 0.0458 | 0.1721 | 0.9092 | 0.8279 | 0.9542 |
| 2019 | Ayacucho | Women | 0.0800 | 0.0461 | 0.1353 | 0.9200 | 0.8647 | 0.9539 |
| 2018 | Cajamarca | Women | 0.1215 | 0.0589 | 0.2339 | 0.8785 | 0.7661 | 0.9411 |
| 2019 | Cajamarca | Women | 0.1472 | 0.0756 | 0.2669 | 0.8528 | 0.7331 | 0.9244 |
| 2018 | Callao | Women | 0.0601 | 0.0311 | 0.1127 | 0.9399 | 0.8873 | 0.9689 |
| 2019 | Callao | Women | 0.0223 | 0.0072 | 0.0672 | 0.9777 | 0.9328 | 0.9928 |
| 2018 | Cusco | Women | 0.0702 | 0.0311 | 0.1508 | 0.9298 | 0.8492 | 0.9689 |
| 2019 | Cusco | Women | 0.0735 | 0.0332 | 0.1550 | 0.9265 | 0.8450 | 0.9668 |
| 2018 | Huancavelica | Women | 0.1608 | 0.0890 | 0.2730 | 0.8392 | 0.7270 | 0.9110 |
| 2019 | Huancavelica | Women | 0.2324 | 0.1467 | 0.3479 | 0.7676 | 0.6521 | 0.8533 |
| 2018 | Huanuco | Women | 0.1344 | 0.0697 | 0.2433 | 0.8656 | 0.7567 | 0.9303 |
| 2019 | Huanuco | Women | 0.0813 | 0.0393 | 0.1606 | 0.9187 | 0.8394 | 0.9607 |
| 2018 | Ica | Women | 0.0413 | 0.0182 | 0.0907 | 0.9587 | 0.9093 | 0.9818 |
| 2019 | Ica | Women | 0.0515 | 0.0235 | 0.1094 | 0.9485 | 0.8906 | 0.9765 |
| 2018 | Junin | Women | 0.0541 | 0.0203 | 0.1361 | 0.9459 | 0.8639 | 0.9797 |
| 2019 | Junin | Women | 0.0351 | 0.0109 | 0.1071 | 0.9649 | 0.8929 | 0.9891 |
| 2018 | La Libertad | Women | 0.0488 | 0.0169 | 0.1328 | 0.9512 | 0.8672 | 0.9831 |
| 2019 | La Libertad | Women | 0.0080 | 0.0011 | 0.0580 | 0.9920 | 0.9420 | 0.9989 |
| 2018 | Lambayeque | Women | 0.0527 | 0.0217 | 0.1223 | 0.9473 | 0.8777 | 0.9783 |
| 2019 | Lambayeque | Women | 0.0547 | 0.0260 | 0.1116 | 0.9453 | 0.8884 | 0.9740 |
| 2018 | Lima | Women | 0.0736 | 0.0448 | 0.1186 | 0.9264 | 0.8814 | 0.9552 |
| 2019 | Lima | Women | 0.0345 | 0.0178 | 0.0659 | 0.9655 | 0.9341 | 0.9822 |
| 2018 | Loreto | Women | 0.0238 | 0.0072 | 0.0758 | 0.9762 | 0.9242 | 0.9928 |
| 2019 | Loreto | Women | 0.0345 | 0.0101 | 0.1106 | 0.9655 | 0.8894 | 0.9899 |
| 2018 | Madre de Dios | Women | 0.0589 | 0.0250 | 0.1325 | 0.9411 | 0.8675 | 0.9750 |
| 2019 | Madre de Dios | Women | 0.0360 | 0.0143 | 0.0875 | 0.9640 | 0.9125 | 0.9857 |
| 2018 | Moquegua | Women | 0.0538 | 0.0252 | 0.1111 | 0.9462 | 0.8889 | 0.9748 |
| 2019 | Moquegua | Women | 0.0432 | 0.0194 | 0.0931 | 0.9568 | 0.9069 | 0.9806 |
| 2018 | Pasco | Women | 0.0406 | 0.0081 | 0.1800 | 0.9594 | 0.8200 | 0.9919 |
| 2019 | Pasco | Women | 0.0993 | 0.0428 | 0.2137 | 0.9007 | 0.7863 | 0.9572 |
| 2018 | Piura | Women | 0.0532 | 0.0192 | 0.1392 | 0.9468 | 0.8608 | 0.9808 |
| 2019 | Piura | Women | 0.0328 | 0.0098 | 0.1044 | 0.9672 | 0.8956 | 0.9902 |
| 2018 | Puno | Women | 0.0950 | 0.0435 | 0.1948 | 0.9050 | 0.8052 | 0.9565 |
| 2019 | Puno | Women | 0.2005 | 0.1034 | 0.3529 | 0.7995 | 0.6471 | 0.8966 |
| 2018 | San Martin | Women | 0.0089 | 0.0012 | 0.0629 | 0.9911 | 0.9371 | 0.9988 |
| 2019 | San Martin | Women | 0.0859 | 0.0446 | 0.1589 | 0.9141 | 0.8411 | 0.9554 |
| 2018 | Tacna | Women | 0.0985 | 0.0523 | 0.1777 | 0.9015 | 0.8223 | 0.9477 |
| 2019 | Tacna | Women | 0.0324 | 0.0112 | 0.0901 | 0.9676 | 0.9099 | 0.9888 |
| 2018 | Tumbes | Women | 0.0312 | 0.0087 | 0.1054 | 0.9688 | 0.8946 | 0.9913 |
| 2019 | Tumbes | Women | 0.0893 | 0.0438 | 0.1737 | 0.9107 | 0.8263 | 0.9562 |
| 2018 | Ucayali | Women | 0.0000 | 0.0000 | 0.0000 | 1.0000 | 1.0000 | 1.0000 |
| 2019 | Ucayali | Women | 0.0869 | 0.0396 | 0.1800 | 0.9131 | 0.8200 | 0.9604 |
