# Supplementary material for: Who is getting screened for diabetes according to body mass index and waist circumference categories in Peru? a pooled analysis of national surveys between 2015 and 2019
Source: PLoS One. 2021 Aug 27;16(8):e0256809. doi: 10.1371/journal.pone.0256809 (PMC8396776; doi:10.1371/journal.pone.0256809)
Supplement: S9 Table — (DOCX) [file pone.0256809.s009.docx]

## **Supplementary table 9: regression models**

|  | **PR** | **95% confidence interval** |
| --- | --- | --- |
| **BMI categories** |  |  |
| Overweight | 1.34 | 1.29-1.38 |
| Obesity | 1.57 | 1.51-1.63 |
| **Year (centred at 2017)** | 1.07 | 1.05-1.10 |
| **BMI categories x year (centred)** |  |  |
| Overweight | 0.96 | 0.93-0.98 |
| Obesity | 0.94 | 0.92-0.97 |
| **Age (years)** | 1.02 | 1.01-1.02 |
| **Sex** |  |  |
| Women | 1.21 | 1.18-1.24 |

Number of observations = 75,333; number of groups (i.e., regions which were included as a random slope) = 25. The regression analysis was conducted in STATA with the following syntax: *meglm test_gluc i.bmi_cat##c.year_c age i.sex || region:, family(poisson) link(log) eform*

|  | **PR** | **95% confidence interval** |
| --- | --- | --- |
| **Waist circumference categories** |  |  |
| Central obesity | 1.63 | 1.35-1.96 |
| **Year (centred at 2017)** | 1.09 | 0.97-1.21 |
| **BMI categories x year (centred)** |  |  |
| Central obesity | 0.95 | 0.85-1.07 |
| **Age (years)** | 1.02 | 1.01-1.02 |
| **Sex** |  |  |
| Women | 1.14 | 1.10-1.19 |

Number of observations = 30,975; number of groups (i.e., regions which were included as a random slope) = 25. The regression analysis was conducted in STATA with the following syntax: *meglm test_gluc i.central_ob2##c.year_c age i.sex || region:, family(poisson) link(log) eform*
