# Supplementary material for: Who is getting screened for diabetes according to body mass index and waist circumference categories in Peru? a pooled analysis of national surveys between 2015 and 2019
Source: PLoS One. 2021 Aug 27;16(8):e0256809. doi: 10.1371/journal.pone.0256809 (PMC8396776; doi:10.1371/journal.pone.0256809)
Supplement: S10 Table — (DOCX) [file pone.0256809.s010.docx]

## **Supplementary table 10: self-reported diabetes diagnosis among people who had a glucose test by BMI category**

| **year** | **BMI category** | **Sex** | **Frequency self-reported diabetes** | **Frequency lower limit** | **Frequency upper limit** |
| --- | --- | --- | --- | --- | --- |
| 2015 | Normal | Men | 0.0308 | 0.0166 | 0.0566 |
| 2015 | Overweight | Men | 0.0309 | 0.0168 | 0.0563 |
| 2015 | Obesity | Men | 0.0505 | 0.0297 | 0.0846 |
| 2015 | Normal | Women | 0.0242 | 0.0115 | 0.0503 |
| 2015 | Overweight | Women | 0.0417 | 0.0266 | 0.0648 |
| 2015 | Obesity | Women | 0.0489 | 0.0343 | 0.0692 |
| 2016 | Normal | Men | 0.0427 | 0.0196 | 0.0908 |
| 2016 | Overweight | Men | 0.0280 | 0.0152 | 0.0510 |
| 2016 | Obesity | Men | 0.0350 | 0.0167 | 0.0720 |
| 2016 | Normal | Women | 0.0407 | 0.0198 | 0.0819 |
| 2016 | Overweight | Women | 0.0449 | 0.0283 | 0.0704 |
| 2016 | Obesity | Women | 0.0523 | 0.0359 | 0.0757 |
| 2017 | Normal | Men | 0.0353 | 0.0144 | 0.0836 |
| 2017 | Overweight | Men | 0.0260 | 0.0135 | 0.0497 |
| 2017 | Obesity | Men | 0.0388 | 0.0191 | 0.0772 |
| 2017 | Normal | Women | 0.0291 | 0.0135 | 0.0615 |
| 2017 | Overweight | Women | 0.0270 | 0.0160 | 0.0449 |
| 2017 | Obesity | Women | 0.0459 | 0.0294 | 0.0710 |

Results are presented as proportions. Multiply by 100 to have percentages. Ov=overweight; Ob=obesity.
